# Super-enhancer-driven TOX2 mediates oncogenesis in Natural Killer/T Cell Lymphoma

**DOI:** 10.1186/s12943-023-01767-1

**Published:** 2023-04-10

**Authors:** Jianbiao Zhou, Sabrina Hui-Min Toh, Tze King Tan, Kalpnaa Balan, Jing Quan Lim, Tuan Zea Tan, Sinan Xiong, Yunlu Jia, Siok-Bian Ng, Yanfen Peng, Anand D. Jeyasekharan, Shuangyi Fan, Soon Thye Lim, Chin-Ann Johnny Ong, Choon Kiat Ong, Takaomi Sanda, Wee-Joo Chng

**Affiliations:** 1 Cancer Science Institute of Singapore, National University of Singapore, 14 Medical Drive, Centre for Translational Medicine, Singapore, 117599 Singapore; 2 Department of Medicine, Yong Loo Lin School of Medicine, National University of Singapore, Singapore, 117597 Singapore; 3 NUS Centre for Cancer Research (N2CR), 14 Medical Drive, Centre for Translational Medicine, Singapore, 117599 Singapore; 4 Division of Cellular and Molecular Research, Lymphoma Genomic Translational Research Laboratory, National Cancer Centre Singapore, 11 Hospital Drive, Singapore, 169610 Singapore; 5 Duke-NUS Medical School, Singapore, 169857 Singapore; 6 Genomics and Data Analytics Core (GeDaC), Cancer Science Institute of Singapore, National University of Singapore, 14 Medical Drive, Singapore, 117599 Singapore; 7 Department of Medical Oncology, the First Affiliated Hospital, Zhejiang University School of Medicine, Hangzhou, 310058 China; 8 Department of Pathology, Yong Loo Lin School of Medicine, National University of Singapore, Singapore, 119074 Singapore; 9 Director’s office, National Cancer Centre, Singapore, 168583 Singapore; 10 Office of Education, Duke-NUS Medical School, Singapore, 169857 Singapore; 11 Department of Sarcoma, Peritoneal and Rare Tumours (SPRinT), Division of Surgery and Surgical Oncology, National Cancer Centre, Singapore, 168583 Singapore; 12 Department of Sarcoma, Peritoneal and Rare Tumours (SPRinT), Division of Surgery and Surgical Oncology, Singapore General Hospital, Singapore, 168583 Singapore; 13 Laboratory of Applied Human Genetics, Division of Medical Sciences, National Cancer Centre, Singapore, 168583 Singapore; 14 SingHealth Duke-NUS Oncology Academic Clinical Program, Duke-NUS Medical School, Singapore, 169857 Singapore; 15 SingHealth Duke-NUS Surgery Academic Clinical Program, Duke-NUS Medical School, Singapore, 169857 Singapore; 16 Institute of Molecular and Cell Biology, A*STAR Research Entities, Singapore, 138673 Singapore; 17 Cancer and Stem Cell Biology, Duke-NUS Medical School, 8 College Road, Singapore, 169857 Singapore; 18 Department of Hematology-Oncology, National University Cancer Institute of Singapore (NCIS), National University Health System (NUHS), 1E, Kent Ridge Road, Singapore, 119228 Singapore

**Keywords:** Super-enhancer, TOX2, Natural Killer/T Cell Lymphoma, RUNX3, PRL-3, Epigenetics, Therapeutic targets

## Abstract

**Background:**

Extranodal natural killer/T-cell lymphoma (NKTL) is an aggressive type of non-Hodgkin lymphoma with dismal outcome. A better understanding of disease biology and key oncogenic process is necessary for the development of targeted therapy. Super-enhancers (SEs) have been shown to drive pivotal oncogenes in various malignancies. However, the landscape of SEs and SE-associated oncogenes remain elusive in NKTL.

**Methods:**

We used Nano-ChIP-seq of the active enhancer marker histone H3 lysine 27 acetylation (H3K27ac) to profile unique SEs NKTL primary tumor samples. Integrative analysis of RNA-seq and survival data further pinned down high value, novel SE oncogenes. We utilized shRNA knockdown, CRISPR-dCas9, luciferase reporter assay, ChIP-PCR to investigate the regulation of transcription factor (TF) on SE oncogenes. Multi-color immunofluorescence (mIF) staining was performed on an independent cohort of clinical samples. Various function experiments were performed to evaluate the effects of TOX2 on the malignancy of NKTL in vitro and in vivo.

**Results:**

SE landscape was substantially different in NKTL samples in comparison with normal tonsils. Several SEs at key transcriptional factor (TF) genes, including *TOX2, TBX21(T-bet), EOMES, RUNX2,* and *ID2*, were identified. We confirmed that TOX2 was aberrantly overexpressed in NKTL relative to normal NK cells and high expression of TOX2 was associated with worse survival. Modulation of TOX2 expression by shRNA, CRISPR-dCas9 interference of SE function impacted on cell proliferation, survival and colony formation ability of NKTL cells. Mechanistically, we found that RUNX3 regulates TOX2 transcription by binding to the active elements of its SE. Silencing TOX2 also impaired tumor formation of NKTL cells in vivo. Metastasis-associated phosphatase PRL-3 has been identified and validated as a key downstream effector of TOX2-mediated oncogenesis.

**Conclusions:**

Our integrative SE profiling strategy revealed the landscape of SEs, novel targets and insights into molecular pathogenesis of NKTL. The RUNX3-TOX2-SE-TOX2-PRL-3 regulatory pathway may represent a hallmark of NKTL biology. Targeting TOX2 could be a valuable therapeutic intervene for NKTL patients and warrants further study in clinic.

**Supplementary Information:**

The online version contains supplementary material available at 10.1186/s12943-023-01767-1.

## Introduction

Extranodal natural killer/T-cell lymphoma (NKTL) is an Epstein-Barr virus (EBV) associated, aggressive non-Hodgkin lymphoma (NHL) that is predominantly localizes to the upper aerodigestive tract but can involve non-nasal sites [1, 2]. The incidence of NKTL shows a significant ethnic and geographic predilection, constituting approximate 10% of NHL in Asia and South America, but only 1% in North America and Western Europe [1, 2]. Combined chemotherapy-radiotherapy is standard treatment for NKTL patients, but often associated with high relapse rate and serious side effects [3]. New drugs, including anti-PD1 antibody pembrolizumab, have been explored [3, 4]. Overall, treatment for NKTL patients remains a challenge in clinic [5, 6]. Novel insight into the molecular mechanisms of this disease would guide the development of effective targeted therapies to improve the survival of NKTL patients, especially for those refractory or relapsed cases [7].

Gene expression profiling studies have reported deregulated signaling pathways underlying the pathogenesis of NKTL, including Janus Kinase/Signal Transducer and Activator of Transcription (JAK/STAT) pathway, PDGF pathway, NOTCH-1 signaling pathway, NFκB pathway [8–11]. Increased expression of *BIRC5* (*Survivin*), *RUNX3*, *AURKA* (*Aurora Kinase A*), and *EZH2* are found in NKTL tumors relative to normal NK cells and they play important roles in the disease progression [12–19]. Furthermore, alterations in epigenetic program have been implicated in the pathogenesis of NKTL [20]. Dysregulated microRNAs (miRNAs) possibly induced by MYC activation affect target pathways relevant to oncogenesis of NKTL [21]. Promoter hypermethylation-mediated silencing of tumor suppressor genes such as *BIM1*, *PRDM1*, *p73*, *DAPK1*, *PTPN6*, and *PTPRK*, have been reported in NKTL patients and cell lines [22, 23]. In addition, somatic mutations have been identified in epigenetic regulator genes, including *ARID1A*, *ASXL3*, *CREBBP*, *KMT2D* (*MLL2*), *KDM6A*, *EP300* and *TET2* in NKTL cases [11, 24].

Enhancer is a region of DNA-regulatory elements that increases the activated transcription of a gene to higher levels via long-range chromatin interaction with its promoter [25]. Super-enhancers (SEs) are defined as large clusters of enhancers in proximity of 12.5 kb with one another [26]. SE regions are often characterized by high level bindings of acetylation of histone H3 lysine 27 (H3K27ac), coactivators and transcription factors (TFs). Common coactivators are mediator complex subunit 1 (MED1), bromodomain containing 4 (BRD4) and EP300 [27–29]. Aberrant assembly and activation of oncogenic SEs have been reported in various solid tumors and hematological malignancies [30–32]. However, the landscapes of SE and their biological functions roles in NKTL remain elusive. In this study, we aim to define the SE landscapes of NKTL for a better understanding of the molecular pathogenesis of NKTL and to identify novel therapeutic targets.

## Materials and methods

### NKTL cell line and patient samples

A panel of NKTL cell lines including NKYS, NK-92, NK-S1, and HANK-1 were used in this study. Detailed characteristics of these NKTL cell lines and their culture conditions were described in supplemental Table S1. Normal NK cells were purchased from Lonza Bioscience (Basel, Switzerland). Primary tumor samples (NKTL4, NKTL9, NKTL10) and their matched normal tonsil tissues were collected at National Cancer Center Singapore with the approval from Institutional Review Board (CIRB Ref: 2018/3084) and informed consent. The clinicopathological characteristics of these 3 patients were presented in supplemental Table S2.

### Super-enhancer peak calling and identification

Nano-chromatin immunoprecipitation followed by sequencing (NanoChIP-seq) was performed on 3 primary NKTL tumor samples and 3 normal tonsil tissues (controls), using polyclonal anti-H3K27ac (Abcam, ab4729) antibody. Library construction and sequencing on the Illumina HiSeq 4000 platform were performed by Exploit Technologies, A*Star (Singapore). Conventional ChIP-seq was conducted on HANK1 and NKYS cell lines using same anti-H3K27ac antibody. ChIP-seq datasets were aligned to the hg19 human genome by Bowtie2 version 2.4.1 with –no-unal and –sensitive parameter. Regions of H3K27ac ChIP-seq peaks were identified by MACS2 2.2.7.1. Constituent enhancers that occurred within 12.5 kb were further stitched together and excluded those that were fully contained within ± 2 kb from TSS for SE identification by Rank Ordering of Super Enhancers (ROSE) with the parameter –s 12,500 and –t 2000. Enhancer regions were plotted in an increasing order based on their H3K27ac ChIP-Seq signal. Enhancers above the inflexion point of the curve were defined as SEs. SEs were assigned to genes with TSS flanking a 50 kb window of the SEs.

### Cell viability assay

CellTiter-Glo® Luminescent Cell Viability Assay (CTG assay, Promega, Madison, WI) was used to determine the cell growth and viability as previously described [33]. Each experiment was in triplicate.

### Lentivirus infection

EGFP-tagged scramble (Scr), TOX2 specific- and RUNX3 specific-shRNAs, FLAG-TOX2 overexpression vector were purchased from VectorBuilder (Chicago, IL, USA). These shRNA sequences were listed in supplemental Table S3. PRL-3-sh1 and -sh2 were previously described [34]. More details of lentivirus infection were described in supplemental Methods.

### RNA-seq and data analysis

Total RNA was extracted using the RNeasy mini kit (Qiagen). RNA-Seq was performed in same 3 pairs of primary NKTL samples and normal tonsils, as well as NKYS treated with scramble shRNA and TOX2-shRNA1 and -shRNA2. The RNA library construction and RNA-sequencing services were provided by Novogene Singapore. Detailed data processing was described in supplemental Methods. Gene Ontology (GO) and pathway analysis conducted by R Bioconductor package GSVA 1.28.0.

### Immunoblotting assay

Cells were lysed with proteinase inhibitor cocktail and phosphatase inhibitor cocktail for 30 min on ice. Immunoblotting was performed using SDS-PAGE followed by protein transfer to PVDF membrane. Primary antibodies were incubated overnight in cold room. Secondary antibodies were incubated for 1 h at room temperature. The following antibodies were used: GAPDH: (Santa Cruz Biotechnology, sc-47724); β-Actin (1:1000, Cell Signaling Technology, CST#4970); TOX2 (Proteintech, 21,162–1-AP), RUNX3 (SC-376591), Cleaved Caspase-3 (CST#9661); Cleaved Caspase-7 (CST#9491), Cleaved PARP (CST#5625). PRL-3 antibody (clone 318) was kindly provided by Dr Qi Zeng (IMCB, A*Star, Singapore).

### Flow cytometric analysis of GPF + cells and cell cycle

The analysis of GFP positive cells was performed on a BD LSR II (Becton Dickinson, USA) flow cytometer, using BD FACSDiva™ software. The cell cycle analyses were carried out using propidium iodide (PI) dye (BD Pharmigen, USA) according to manufacturer’s instructions.

### ChIP-PCR

ChIP followed by PCR (ChIP-PCR) analysis was performed on NKYS cells to evaluate TOX2 binding in the promoter region of PTP4A3. Rabbit polyclonal antibody to TOX2 (21,162–1-AP; Proteintech, USA) or its respective IgG isotype control was used for ChIP. Primers for amplification of the regions with or without consensus TOX2 binding sequence in the promoter of PTP4A3 were included in supplemental Table S3.

### Enhancer luciferase assay and site-directed mutagenesis

Selected enhancer regions within TOX2-SE, as well as mutant RUNX3 binding motif were cloned into the PGL4.26 vector (primer sequences provided in supplemental Table S3 and their activity was assayed using a dual luciferase reporter assay (Promega). A region outside TOX2-SE with low H3K27Ac signal was cloned as negative control (TOX2-eNC). Site-directed mutagenesis for RUNX3 binding motif was performed using QuikChange II Site-Directed Mutagenesis Kit (Agilent, Santa Clara, CA) following the manufacturer’s instructions. PCR-amplified enhancer candidates were inserted downstream from the firefly luciferase gene at the KpnI and NheI site in the pGL4.26 vector and cotransfected with a Renilla luciferase encoding plasmid (pGL4.75) into HEK293T cells on 96-well plates. Luciferase activity (firefly/Renilla) was measured on the Glomax 20/20 Luminometer (Promega) following the manufacturer's protocol.

### CRISPR/dCas9-KRAB interference

To generate the dCas9-KRAB-T2A-mCherry expression vector, the GFP expression cassette of the dCas9-KRAB-T2A-GFP lentiviral vector (Addgene plasmid #71,237) was replaced by mCherry sequence. sgRNAs targeting the TOX2 enhancer region were designed using the online tool (http://crispor.tefor.net/). The sgRNA oligos were synthesized, annealed and cloned into inducible gRNA vector with GFP reporter FgH1tUTG (Addgene plasmid # 70,183) which were *BsmBI* digested and dephosphorylated. The sgRNA sequences were listed in supplemental Table S3. Lentiviruses were produced by co-transfecting dCas9-KRAB-T2A-mCherry plasmid with pMDLg/pRRE, pRSV-Rev, and pMD2.G into HEK293T cells using X-tremeGENE HP DNA Transfection Reagent (Roche). Lentiviral supernatant was harvested 72 h post-transfection. NKYS cells were infected with dCas9-KRAB-mCherry-expressing lentivirus in the presence of polybrene (Millipore) followed by sorting for mCherry positive population using FACSAria Flow Cytometer (BD Biosciences). The inducible sgRNA lentiviruses were then transduced into NKYS cells with stable dCas9-KRAB-mCherry expression. Doxycycline was added at a concentration of 1 µg/ml following infection to allow for repression of enhancer activity. Control cells were treated with DMSO.

### Multiplex immunofluorescence analysis of validation study cohort

Existing tissue microarray (TMA) samples of patient diagnosed with NKTL between 1992 and 2017 (*n* = 42) in the Department of Pathology, National University Hospital (NUH) were used for multiplexed immunofluorescence (mIF) as previously described [35]. This study was approved by the National Healthcare Group Domain Specific Review Board B (2009/00212). The clinicopathological characteristics, therapeutic regime and outcome of these patients were described in supplementary Table S9.

We developed an automated mIF staining protocol for CD3/PRL-3/TOX2/RUNX3 panel through Leica Bond Max (SN: M211523) based on a published protocol [35]. CD3 was used for NKTL tumor cell marker. Traditional DAB immunohistochemical staining was used to optimize the staining parameters, for each antibody separately using the Leica Biosystems Bond Polymer Refine Detection Kit (DS9800). For mIF staining, briefly, the slides were baked and dewaxed followed by heat induced epitope retrieval (HIER) at 100 °C in antigen retrieval buffer for 20 min. The slides were then peroxidase blocked (only for the 1^st^ marker) for 10 min. Markers were prepared in DAKO antibody diluent followed by the polymeric HRP-conjugated secondary antibody (DS9800) and opal fluorophore-conjugated TSA (Akoya Bioscience) at 1:100 dilution before dispensing to the slide sequentially. Slides were rinsed with 1 × washing buffer after each step. After staining the opal fluorophore for the 1^st^ marker, slides were heated at 100 °C again to strip the primary and secondary antibodies bound to the tissue for labelling of the next marker. These steps were repeated until all remaining markers were labelled.

The antibody sequence, dilution, antibody-opal pairs and antigen retrieval conditions for the multiplex staining were as follow: TOX2 (ProteinTech, 21,162–1-AP, dilution 1:500–20 min, HIER solution 1–20 min)– Opal 520; RUNX3 (Santa Cruz, sc-376591, dilution 1:500–20 min, HIER solution 2–20 min) – Opal 570; PRL-3 (ProteinTech, 15,186–1-AP, dilution 1:100–30 min, HIER 1 solution -20 min)– Opal 540; CD3 (DAKO, A4052, dilution 1:200–20 min, HIER solution 2–20 min) – Opal 620. Finally, DAPI (Akoya Biosciences, FP1490) at 1:10 dilution was added as a nuclear counterstain. Slides were imaged using Vectra 2 Single Slide (Akoya Biosciences, S/N: VT1447N8001). The component images for each marker were exported through Inform software (Akoya Biosciences, Version 2.4.8). The staining signal for each marker for the mIF images were unmixed through Inform (Akoya Biosciences, Version 2.4.8). After unmixing the staining signal, the component images for each marker were also exported through Inform software. All these component images were imported to Visiopharm (Denmark) for image analysis. A purchased APP “Nuclei Detection, AI (Fluorescence)” was used for cell segmentation. The deep learning APP for CD3 phenotyping were trained on multiple CD3 positive/negative labelling images with a variety of CD3 staining intensity. Data were then exported from Visiopharm for analysis.

### In vivo xenograft model

For the human NKTL cell line xenograft model, we used female NOD.Cg*-Prkdc*^*scid*^* Il2rg*^*tm1Wjl*^/SzJ, NGS mice (6—7 weeks old), purchased from The Jackson Laboratory (Bar Harbor, ME, USA) through InVivos (Singapore). The animals were maintained in specific pathogen-free conditions. Ten million of NK-S1-scramble and NK-S1-TOX2-sh1 cells were mixed with Matrigel (50%) and subcutaneously injected into each side of loose skin between the shoulder blades and the hind leg of NGS-recipient mice (*n* = 5), respectively. The length (L) and width (W) of the tumor were measured with calipers every 2 -3 days, and tumor volume (TV) was calculated as TV = (L × W^2^)/2. At the end of experiments, mice were euthanized and tumors were dissected. The protocol is reviewed and approved by the Institutional Animal Care and Use Committee (IACUC) in compliance to the guidelines on the care and use of animals for scientific purpose (protocol number: R18-1254).

## Statistical analyses

Prism 9.0 software (GraphPad Software, San Diego, CA, USA) was used to perform statistical analysis and make graphs. Survival curves were constructed and compared with the Kaplan–Meier method. Chi-square test was performed to analyze the categorical correlation. Student's t-test and Mann–Whitney test were used to analyze parametric and nonparametric variables, respectively. Statistical significance was achieved with a p-value of less than 0.05.

## Results

### Mapping super-enhancer landscape in NKTL

**Fig. 1:**
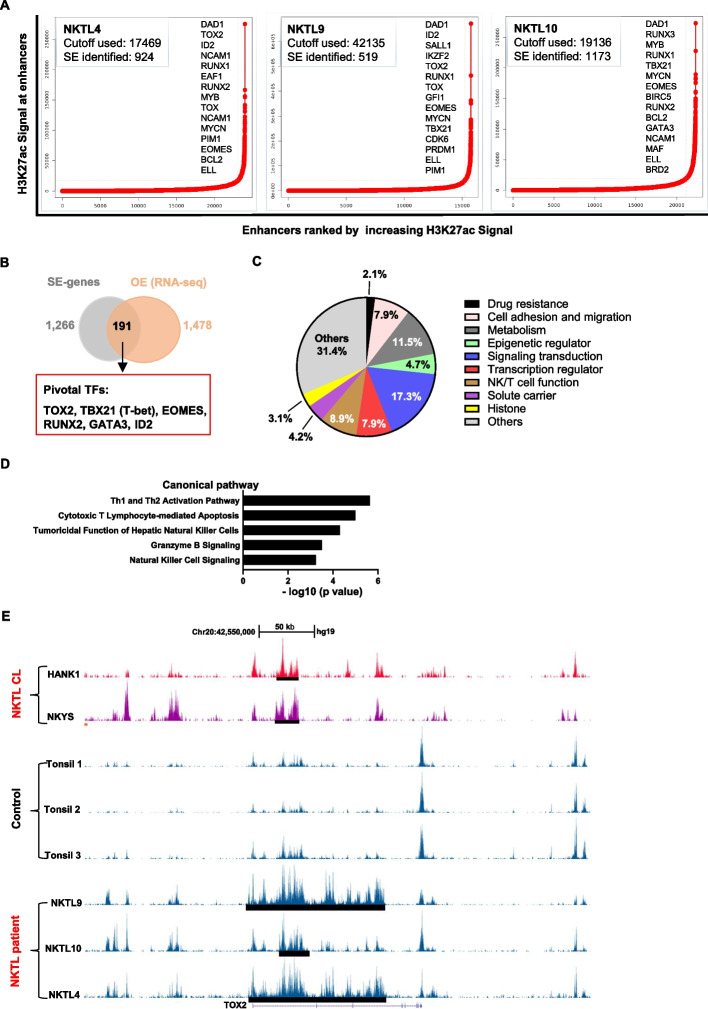
The Super enhancers landscape of primary NKTL patient samples, NKTL cell lines and the controls. **A** Enhancer regions of in 3 primary NKTL patients. Enhancers were ranked by increasing H3K27Ac signal, and enhancers above the inflection point of the curve were defined as SEs, and the number of SEs was shown for each sample. Examples of SEs associated genes found in at least two primary MM cases were also presented. **B** Schematic diagram of the selection criteria for high-confident candidate SE-associated genes. **C** The list of final 191 SE-associated genes selected according to the criteria shown in (**B**) was classified into different function group. **D** NKTL-SE genes were enriched in multiple signaling pathways related to NK cell function. **E** Track view of H3K27ac ChIP-seq density profile centered at the *TOX2* gene loci of NKTL cell line HNAK1 and NKYS (top panel), 3 tonsil controls (middle panel) and 3 primary NKTL patient samples (lower panel). Locations of the SEs regions were marked by black bars

To understand the epigenetic regulations in NKTL, we carried out ChIP-seq using antibodies against H3K27ac on 3 NKTL tumor, 3 normal tonsil control samples and 2 NKTL cell lines (HANK1 and NKYS). H3K27ac is a major active enhancer-associated chromatin modification and significant clustering of H3K27ac is a distinct feature of SE. After the initial quantification and alignment of the reads to the human genome, we first performed the global analysis for H3K27ac histone modification (Fig. 1A). To map the SE landscapes in NKTL, we performed the ROSE analysis, which identifies super-enhancers based upon H3K27ac ChIP-seq data. A total of 1266 SEs were identified in more than 2 out of 3 primary NKTL tumors but not in their normal tonsils (Fig. 1B). The complete list of the SE-genes was presented in supplemental Table S4.

As SEs often drive high transcriptional outputs, we hypothesized that combining SE profiles with gene expression data derived from same samples would allow us to pinpoint novel oncogenes that are critically involved in NKTL pathogenesis. To this end, we filtered genes that were associated with super-enhancers and significantly overexpressed in NKTL tumors compared to normal tonsils. RNA-seq revealed overexpression of 1478 genes (false discovery rate < 0.001, log2 fold change ≥ 1, supplemental Table S4). Using this rigorous strategy, we pinned down a list of 191 SE-associated genes that are over-expressed in NKTL (from this point known as NKTL-SE genes) which is worthy of further investigation (supplemental Table S4). The list of genes could be classified into different function groups, including Drug resistance, Cell adhesion and migration, Metabolism, Epigenetic regulator, Signaling transduction, Transcription regulator, NK/T cell function, Solute carrier, Histone and Others (Fig. 1C). Signaling pathway analysis revealed that these NKTL-SE genes were highly enriched in key pathways related to NK/T cell function and signaling (Fig. 1D). However, several known pathways closely related to different molecular subtype of NKTL such as JAK-STAT pathway in TCR-negative NKTL, RAS-MAPK pathway in TCR-positive NKTL, and others, did not show high rank in our pathway analysis [11], Xiong J and colleagues developed a novel algorithm based on a quantitative gene expression metrics of NK-cell and T-cell associated genes to categorize patients into NK-cell origin and T-cell origin [11]. We intend to categorize NK/T origin of patients from our RNA-seq data. Then using a two-sample Kolmogorov–Smirnov based method developed in house [36], and the signatures from Xiong’s study [11], we estimated the score of NK-cell origin or T-cells origin. Consistent with the dot plot shown, all 3 NKTL cases were estimated to originate from NK cells (supplemental Figure S1). Pivotal TFs known to regulate NK cell development and function were highly enriched in the list: TOX2, TBX21(T-bet), EOMES, RUNX2, GATA3, and ID2. Notably, TOX2 is also a member of a small subfamily of proteins (TOX, TOX3, and TOX4) that share almost identical high mobility group (HMG)-box sequences. TBX21 and EOMES are members of T-box protein family. We then scrutinized the SE constituents of these 3 genes (TOX2, TBX21 and EOMES) and found that only TOX2 harbored remarkably high SE peaks specifically in all NKTL samples and two NKTL cell lines, in contrast, only background signals were presented in normal tonsil tissues (Fig. 1E and supplemental Figure S2).

## TOX2 is overexpressed in NKTL and associated with poor survival

**Fig. 2:**
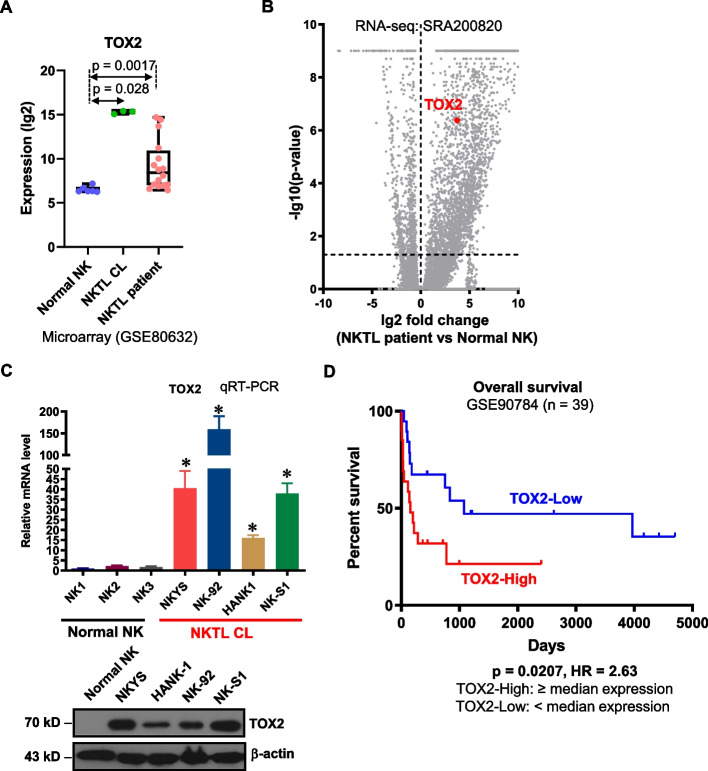
The expression and prognostic value of TOX2 in NKTL. **A** Expression (lg2) level of *TOX2* in a collection of normal NK cells, NKTL cell line and NKTL patient samples derived from a microarray dataset in Gene Expression Omnibus (GEO) database (accession number: GSE80632). **B** Volcano plot demonstrating gene expression level in a collection of normal NK cells and NKTL patient samples derived from an RNA-seq dataset deposited in the Sequence Read Archive (SRA) database, under the accession code SRA200820. Y-axis represents p value (lg10). X-axis indicates the fold change (lg2) of genes differentially expressed between normal NK cells (left) and NKTL patient samples (right). *TOX2* was labelled. **C** Quantitative RT-PCR of *TOX2* gene expression in 3 normal NK cell samples and NKTL cell line NKYS, NK-92, HANK and NK-S1 (upper panel). The expressions of TOX2 gene were normalized to GAPDH level (internal control) for each sample and are presented as relative fold changes (*n* = 3, mean ± SD). *p < 0.01 for comparison of NKTL cell lines vs. normal NK cells. Western blotting analysis of TOX2 protein in one normal NK cell sample and 4 NKTL cell lines (lower panel). β-actin was used as a loading control. This result is representative for three independent biological replicates. **D** Utilizing data (GSE90784) from GEO database, *TOX2* expression was categorized into TOX2- High (≥ 50%) and TOX2-Low (< 50%) group. Kaplan–Meier survival curves were constructed for NKTL patients based on *TOX2* expression levels (TOX2-Low vs TOX2-High). Significance (*p*) was evaluated by Log-rank test. HR: hazard ratio

The presence of TOX2-SE in all NKTL samples, but not in normal controls suggests that this gene is specifically and highly activated in NKTL cells. In fact, TOX2 expression was significantly higher in the primary NKTL samples and cell lines compared with normal NK cells (microarray dataset GSE80632 and RNA-seq datasets SRA200820) (Fig. 2A, 2B). qRT-PCR and Western blot analysis further confirmed the overexpression of TOX2 mRNA and protein in NKTL cell lines relative to normal NK cells (Fig. 2C).

In order to establish the clinical significance of TOX2 in NKTL, we conducted survival analysis on our published gene expression dataset (GSE90784). Most importantly, a higher expression of TOX2 was associated with worse overall survival (Log rank p value: 0.021, hazard ratio: 2.63), demonstrating its prognostic significance (Fig. 2D). Taken together, these data argue for the implication of TOX2 in the pathogenesis of NKTL.

### TOX2 mediates NKTL cell growth, proliferation and colony formation

**Fig. 3:**
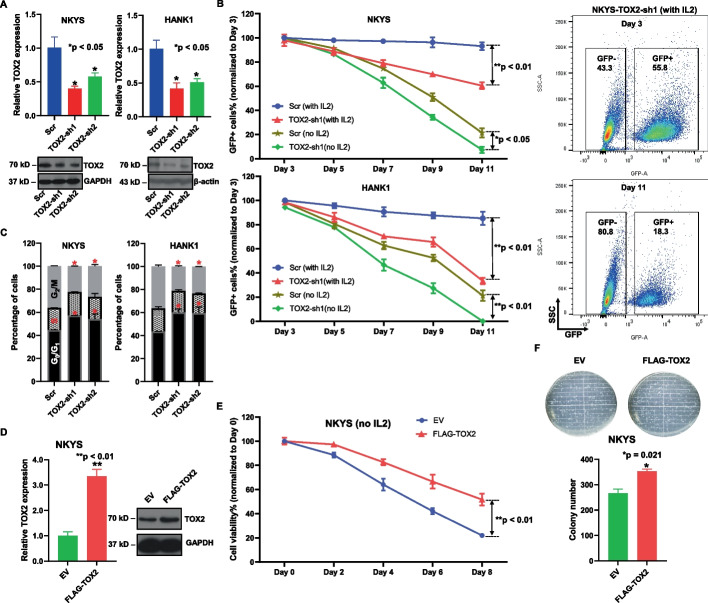
Oncogenic properties of TOX2 in NKTL cells. **A** NKYS and HANK1 cells were infected with either scramble shRNA, or TOX2-sh1 or TOX2-sh2 tagged with green fluorescent protein (GFP) for 3 days, then subjected to mRNA and protein extraction. Quantitative RT-PCR (upper panel) and immunoblotting analysis (lower panel) of TOX2 transcript and protein level in these populations. Three independent experiments were conducted. For qRT-PCR analysis, data were normalized to GAPDH level (internal control) for each sample and are expressed as the fold change vs scramble control population (mean ± SD). *p < 0.05. For immunoblotting analysis, GAPDH and β-actin were used as loading controls in NKYS cells and HANK1 cells, respectively. Representative blotting images were shown. **B** Flow cytometric analysis of the percentage of GFP + cells post-infection of NKYS and HANK1 cells. The quantification started at day 3 post-infection at 2-day intervals up to day 11. The percentage of GFP + cells at day 5, 7, 9, 11 was normalized to day 3, respectively. Two sets of cell culture medium with or without human IL-2 (10 ng/ml) were used. Each data point was representative of three biological replicates (mean ± SD). *p < 0.05; **p < 0.01. Representative FACS plots show NKYS cells infected with TOX2-sh1 lentivirus at day 3 and day 11. **C** Cell cycle analysis of NKYS and HANK1 cells infected with either scramble shRNA or TOX2-sh1 or TOX2-sh2 lentivirus. These cell cycle experiments were triplicated and presented in mean ± SD. *p < 0.05. **D** Quantitative RT-PCR of *TOX2* gene expression in NKYS cells transduced with either empty vector (EV) or FLAG-TOX2 overexpression vector. These data show mean ± SD of 3 independent experiments. **p < 0.01 (left panel). Western blot analysis of TOX2 protein level in EV-NKYS cells and FLAG-TOX2-NKYS cells. GAPDH was used as loading control (right panel). **E** Quantification of the percentage of GFP + subpopulation among NKYS-EV and NKYS-FLAG-TOX2 cells at 2-day interval up to day 8. Human IL-2 was removed from culture medium. This experiment was repeated 3 times. **F** TOX2 increases colony formation of NKTL cells. Representative images of colony formation captured from NKYS-EV and NKYS-FLAG-TOX2 cells (upper panel). The numbers of colony in 10 random field was illustrated in mean ± SD (lower panel). These data were from three independent experiments. *p = 0.021

After demonstrating its overexpression and prognostic value, we proceeded to further study TOX2’s functional roles in NKTL. Two individual TOX2 specific-shRNAs tagged with GFP were transfected into NKYS and HANK1 cells. qRT-PCR and immunoblotting analysis confirmed the decreased TOX2 mRNA and protein induced by TOX2-sh1 and –sh2 compared to scr-shGFP (control) (Fig. 3A). To assess whether TOX2 knockdown inhibits the growth of NKTL cells, we quantified the GFP + % TOX2-sh1-transduced NKYS cells at 2-day intervals starting 3 days post transduction. Because the growth of NKYS and HANK1 cells requires human cytokine IL-2, we performed two sets of experiments with or without IL-2 in culture medium in parallel at day 3. We observed a markedly decreased GFP + % cells compared with scr-shGFP-transduced cells in both settings (Fig. 3B**)**, however, the difference was more remarkable in medium containing IL-2. These results indicate that silencing TOX2 imposes a strong negative selection pressure on NKTL cell growth (Fig. 3B). Cell cycle analysis revealed that inhibition of TOX2 had impact on cell cycle distribution in NKYS and HANK1 cells. Compared with control samples, TOX2-sh1- and –sh2-treated samples had a significant increase in G_0_/G_1_-phase populations (Fig. 3C). Next, we overexpressed TOX2 in NKYS cells, and confirmed that TOX2 mRNA and protein were increased in NKYS cells overexpressing FLAG-TOX2 plasmid relative to empty vector (EV) control cells (Fig. 3D). This pair of cells were then cultured with or without the addition of IL-2 cytokine and CTG assay were done at different time points. The result showed that there is no significant difference in cell viability between these two lines in presence of IL-2 (Data not shown), however, NKYS-TOX2 cells maintained significantly higher cell viability than NKYS-EV in the culture without IL-2 (Fig. 3E). These data suggested that TOX2 confers growth advantage for NKTL cells in absence of cytokines. To evaluate the effect of TOX2 on the clonogenicity of NKTL cells, colony growth was determined by CFU assay in NKYS-TOX2 and –EV cells. The number of CFU was significantly increased in NKYS-TOX2 cells compared with NKYS-EV cells (Fig. 3F, *p* = 0.021). The results demonstrate that TOX2 is effective in enhancing the clonogenic capacity of NKTL cells.

### TOX2 expression is driven by super-enhancer

**Fig. 4:**
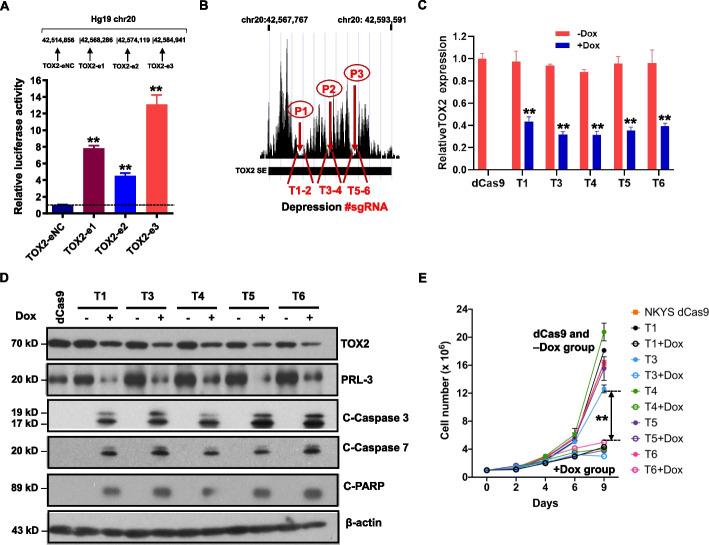
Functional importance of TOX2-SE in NKTL cells. **A** Enhancer activity was identified in a reporter assay for TOX2-eNC (a low H3K27Ac region outside of TOX2-SE on Chr20), TOX2-e1, TOX2-e2 and TOX2-e3 regions, respectively. The position of each region on chr20 was indicated (not in size scale). Enhancer activity is expressed as relative fold change of TOX2-SE regions (-e1, -e2, -e3) vs control region (-eNC). Three biologically independent assays were performed. Error bars represent SD. **p < 0.001. **B** A schematic diagram of the pairs of sgRNAs designed to target 3 valley bases (P1, P2 and P3) on H3K27Ac track of the SE region of *TOX2*. Two pairs of sgRNAs were used to direct the dCas9-KRAB transcription repression system to target 2 sites of each valley base (T1-2 for P1; T3-4 for P2; T5-6 for P3). **C** Decreased mRNA expression of *TOX2* target gene after activation of pairs of sgRNAs (T1, T3-6) guided dCas9–KRAB repression system targeting the TOX2-SE region (*n* = 3 biologically independent samples of NKYS cells). dCas9: stable NKYS-dCas9 cells without pairs of sgRNAs. Dox: doxycycline. Student’s t-test was applied for all statistical comparisons of TOX2 expression in cells + Dox versus -Dox (**p < 0.01). **D** TOX2, PRL-3, and apoptosis-related proteins were analyzed by Western blot in NKYS-dCas9 cells after transfected with pairs of sgRNA in condition of + Dox or -Dox. Detection of β-actin protein was used as an internal loading control. Three independent experiments were conducted and representative blot images were shown. **E** Cell proliferation assays with different pairs of sgRNA transfected NKYS-dCas9 cells with or without Dox induction. The number of cells over 9 days was recorded under each condition as indicated. Data of three biological replicates (mean ± SD) were used to construct these growth curves. **p < 0.001 for the different of -Dox versus + Dox group

To investigate correlation between super-enhancer activity and H3K27Ac signals on TOX2-SE identified, we cloned 3 different enhancer regions and examined their enhancer activity in an enhancer reporter assay. Cloned SE regions significantly increased luciferase signal (enhancer activity), while a cloned region outside of SE with background H3K27Ac signal failed to do so (Fig. 4A). This finding suggested that this TOX2-SE has regulatory activity.

Next, we assessed whether the TOX2-SE is functional and causative for the TOX2 dysregulation in NKTL. To this end, we synthesized 6 pairs of sgRNAs T1 to T6 targeting the SE peaks spanning the ~ 0.3-Mb genomic region and transduced into the NKYS line stably expressing dCas9-KRAB (Fig. 4B, supplemental Table S5). T2 pair was excluded from analysis due to unsuccessful lentiviral packaging. All the remaining 5 pairs enabled effective KRAB-dCas9-mediated epigenetic silencing and reduced TOX2 expression on both mRNA (Fig. 4C) and protein level (Fig. 4D) upon doxycycline (Dox) induction. The inhibition of SE activity in sgRNAs-CRISPR/dCas9-transfected cells led to a significant decrease in cell growth (Fig. 4E). Furthermore, we also observed that expression of several active apoptosis markers such as cleaved caspase-3, cleaved caspase-7, and cleaved PARP, were increased in Dox-treated cells, compared to cells without Dox treatment or without sgRNAs (dCas9 only) (Fig. 4D). These findings supported the regulatory activity of TOX2-SE on the elevated expression of TOX2 and reflected the importance of TOX2-SE on the downstream functional effect of TOX2.

### Genetic inhibition of TOX2 reveals key TOX2-regulated oncogenes required for NKTL cell survival

**Fig. 5:**
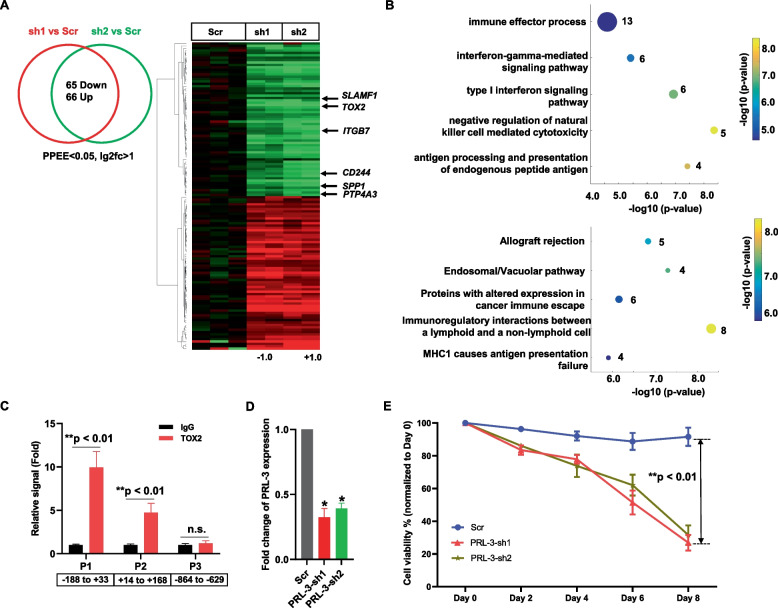
Genetic inhibition of TOX2 in NKTL cells. **A** Overlap analysis (left panel) and heatmap (right panel) of genes that were differentially expressed induced by knockdown of NKYS cells. Here, FDR of 0.1 was used as a cutoff. Significant gene expression changes are defined by DESeq2 algorithm with fold change ≥ 2 and adjusted *p* < 0.05. Selected 6 genes including *TOX2* were highlighted on the heatmap. **B** Gene ontology enrichment analysis (upper panel) and pathway analysis (lower panel) of TOX2-regulated genes revealed by RNA-seq analysis. **C** TOX2 occupancy on TOX2 binding sites in the PRL-3 (PTP4A3) promoter was examined by ChIP using anti-TOX2 antibody with IgG as negative control. ChIP-qPCR was conducted using primers flanking TOX2 binding sites in PRL-3 promoters (P1 and P3). A region without TOX2 binding site (P3) was used as a control. The occupancy of TOX2 on these sites were calculated as percentage of the respective input DNA concentration and expressed as relative signal after normalized against the IgG samples (set as 1). Values are shown as mean ± SD of four independent experiments. **, significantly higher (*p* < 0.01) than the respective IgG samples. n.s., not significant. Negative and positive numbers indicate the regions relative to the TSS of PRL-3. **D** NKYS cells were transfected with scramble shRNA (Scr) or PRL-3-sh1, -sh2. Efficacy of *PRL-3* silencing measured by qRT-PCR. Data were normalized to GAPDH level (internal control) for each sample and are expressed as the fold change relative to scramble control population (mean ± SD) *p < 0.05. **E** Cell viability was assessed by CTG assay every 2 days up to day 8. The percentage of cell viability of day 2, 4, 6, 8 was compared with day 0 as baseline (100%). Data shown are the average of 3 independent experiments and each experiment was done in triplicate. **p < 0.01, significant difference between Scr group and PRL-3-sh group

To gain insight into the role of TOX2 in pathogenesis of NKTL, we conducted a transcriptomic analysis of NKYS cells expressing Scramble shRNA or TOX2-sh1 or TOX2-sh2. Using twofold as cut-off level (FDR < 0.05, *p* value < 0.05), 65 genes showed decreased expression and 66 genes had increased expression in both NKYS-TOX2-sh1 and -TOX2-sh2 expressing cells compared to NKYS-Scramble shRNA cells (Fig. 5A, supplemental Table S5). In addition to *TOX2* gene, metastatic oncogene *PTP4A3* (Protein Tyrosine Phosphatase 4A3, also known as PRL-3), *SPP1*, *ITGB7*, *SLAMF1* (*CD150*) and *CD244* (*SLAMF4*) genes were downregulated in TOX2-sh-treated cells. GO term analysis revealed that genes involved in immune response and regulation of NK cell activity showed the most significant change (Fig. 5B). Pathway analysis identified top five canonical pathways, including Allograft rejection, Endosomal/Vacuolar pathway, Proteins with altered expression in cancer immune escape, Immunoregulatory interactions between a lymphoid and a non-lymphoid cell, and MHC1 causes antigen presentation failure (Fig. 5B). We also used GeneMANIA online program to interrogate the 65 downregulated genes and constructed gene—gene interaction networks [37]. This analysis also revealed that TOX2 sat on the top of the network. A physical interaction of sub-network, comprising MHC family members was formed (supplemental Figure S3). It appears that SLAMF1 might play a role in regulation of this cluster of MHC family genes. It has been reported that SLAMF1 expression is restricted to some hematologic malignancies including cutaneous T-cell lymphomas, a few types of B-cell non-Hodgkin's lymphoma, chronic lymphocytic leukemia, Hodgkin's lymphoma, and multiple myeloma [38]. Thus, it is potentially viable approach to target NKTL cells with anti- SLAFM1 antibody or measles virus (MV) oncolytic therapy because SLAFM1 serves as a cellular receptor for wild type as well as vaccine strains of MV [38].

To examine the mechanisms whereby TOX2 regulates target genes, we interrogated a publicly available dataset, TOX2 ChIP-seq of a neuroblastoma cell line, SK-N-SH (ReMap2022, Experiment ID: ENCSR226NRS) on these 65 genes for their genomic regions overlapping with TOX2 binding (ChIP-seq peak). These analyses identified 23 TOX2-bound genomic sites (supplemental Table S6). TOX2 was enriched at promoter-transcription start sites (TSS) of *PTP4A3* and *LIMCH1* gene, while all other binding occurred at 3’-UTR (untranslated region), intergenic, or intron sites (supplemental Table S6). Next, to determine whether PTP4A3 is a direct target gene of in vivo, we identified two stretches of regions harboring consensus TOX2 binding motif (VSSSGVVGCG) in PTP4A3 promoter (supplemental Table S7). Next, we conducted qPCR using two pairs of primers covering these TOX2 binding motifs in the ChIP DNA extracted from NKYS cells. Our analysis confirmed genomic TOX2 binding within a region spanning the approximate -200 bp to + 200 bp relative to the PTP4A3 TSS (Fig. 5C, supplemental Table S7). Importantly, in above-described CRISPP-interference experiments, we observed decreased PRL-3 protein level in parallel to reduced TOX2 expression upon Dox-induction (Fig. 4D).

PRL-3 was chosen for further functional study because of its widely reported oncogenic role in the literature, while the function of LIMCH1 appears inconsistent in different type of cancers. Therefore, NKYS cells were stably transduced with lentivirus-mediated shRNA targeting PRL-3 (-sh1, -sh2) or Scramble shRNA (Scr). The knockdown effect of PRL-3-sh1 and -sh2 was confirmed by qRT-PCR analysis (*p* < 0.05, Fig. 5D). Compared with the NKYS-Scr control cells, NKYS-PRL-3-sh1 and -sh2 cells exhibited significantly decreased viability (*p* < 0.01; Fig. 5E). These results indicate that PRL-3 is regulated by TOX2 and plays an important role in TOX2-mediated oncogenesis in NKTL cells. Taken together, these data reveal TOX2-regulated pathways, networks in which PRL-3 is a key downstream oncogene.

### RUNX3 binds on TOX2-SE and promotes TOX2 gene transcription

**Fig. 6:**
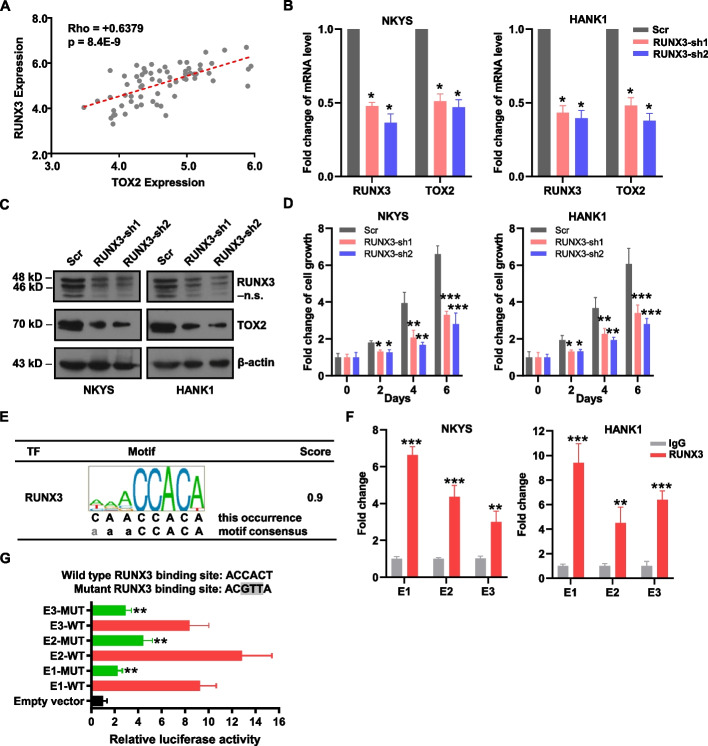
RUNX3 bound to the SE and activated the expression of TOX2. **A** Correlation between *RUNX3* expression with *TOX2* expression in NKTL patients from GEP dataset: GSE90784. A significant positive correlation was determined by Pearson's p value = 8.39E-09, and R = 0.64. **B, C** The mRNA (B) and protein (C) levels of RUNX3 and TOX2 were detected by qRT-PCR and Western blot analysis upon transfection with two different pairs of RUNX3-shRNA (RUNX2-sh1, -sh2) or the scramble shRNA (Scr) in NKYS and HANK1 cells. GAPDH was measured for data normalization (B) and β-actin was used as the loading control (C). n.s.: non-specific band produced by anti-RUNX3 antibody (A-3 clone, sc-376591) in addition to specific bands at 48, 46 kD. All these results were representative for three independent biological replicates. **D** Relative cell growth was measured in NKYS and HANK1 cells transduced with RUNX3-shRNAs or Scr-shRNA. For each condition, cell number was counted at day 2, 4, and 6, then converted to fold change relative to the starting number at day 0. Same number of cells were seeded at day 0 and comparison was made at indicated time points for relative fold changes of cells transduced with RUNX3-shRNA versus Scr. Three biologically independent experiments were performed (mean ± SD). *p < 0.05, **p < 0.01, ***p < 0.001. **E** RUNX3 binding site locates within the TOX2-SE loci. **F** ChIP-PCR confirmed the interaction between RUNX3 and SE region of TOX2 in NKYS and HANK1 cells. Data are expressed as fold change of RUNX3 antibody-IP vs IgG control-IP. Data are representative of 3 independent IPs. Error bars indicate SD. **p < 0.01, ***p < 0.001 by two-sample, two-tailed t-test compared with the control. **G** Indicated vectors were transiently transfected into 293 T cells, and luciferase activity was measured using a Dual-Luciferase system. Firefly luciferase activity was normalized to co-transfected Renilla luciferase and calculated as relative fold change to pGL4.26 empty vector. Data shown represent means ± SD of three independent experiments. **p < 0.01, compared with each RUNX3-WT group (E1, E2, and E3), respectively. WT: wild type; MUT: mutant

Runt-related transcription factor (RUNX) proteins, including RUNX1, RUNX2 and RUNX3, belong to a transcription factors family shared conserved DNA-binding sequences-PPPYP (RUNX domain) [39]. RUNX1 and RUNX3 are important for hematopoietic cell differentiation, RUNX2 is essential for osteogenesis. RUNX3 also regulates growth of gastric epithelial cells [40]. During latent infection of EBV, RUNX3 is a direct target of the viral transcription factor EBNA2 and the induced RUNX3 protein binds to the conserved RUNX binding site near the TSS of RUNX1 P1 promoter, leading to the repression of RUNX1. Expression of RUNX3 and repression of RUNX1 are required for efficient proliferation of B cells immortalized by EBV [41, 42]. Indeed, we confirmed RUNX3 expression is significantly higher in NKTL cell lines and NKTL patient tumor samples when compared to normal NK cells (*p* = 2.8E-05 and 1.1E-04, respectively). In contrast, RUNX1 expression is lower in NKTL cell lines relative to normal NK cells (*p* = 0.036), but its level is not statistically different among NKTL patient tumor samples and normal NK cells (*p* = 0.099) (supplementary Figure S4). Taken together, these data imply that RUNX3, but not RUNX1, is potentially relevant in NKTL disease. Furthermore, we previously reported that RUNX3 was overexpressed in NKTL with functional oncogenic properties [17]. These rationales led us to further characterize RUNX3 in NKTL. Next, we performed correlation analysis on publicly available GEP dataset (GSE90784) and identified RUNX3 expression was significantly correlated with TOX2 (Fig. 6A, Pearson's R = 0.64; Pearson's *p* value = 8.39E-09). To study whether RUNX3 could regulate TOX2 transcription, we next knocked down RUNX3 using two independent shRNAs. A significant decrease in TOX2 mRNA and protein expression was observed (Fig. 6B and 6C) in both NKYS and HANK1 cells, implying that *TOX2* could be a downstream target gene of *RUNX3*. Importantly, inhibition of cell growth was confirmed in both cell lines infected with RUNX3-shRNA lentiviral particles (Fig. 6D). To confirm the specificity of TOX2 as a target of RUNX3, we created co-transduced NKYS cells expressing RUNX3-sh1 with FLAG-EV or FLAG-TOX2 construct. NKYS cells depleted of RUNX3 with the FLAG-EV had a cell proliferation rate that was reduced by up to 55%, and the effect of this knockdown was completely reversed by ectopic expression of FLAG-TOX2 (supplemental Figure S5).

We further investigated if this positive correlation is due to RUNX3 binding to the SE of *TOX2* gene. Analyzing ChIP-seq data for motif discovery via Factorbook developed by the ENCODE consortium [43], several binding sites of RUNX3 including E1, E2, and E3, were located within the TOX2-SE region (Fig. 6E and supplemental Table S8). Notably, we performed ChIP-qPCR, confirming that the ChIP enrichment signal of RUNX3 was specific within SE region of TOX2 (Fig. 6F and supplemental Table S8). We hypothesized that the consensus recognition motif ACCACA is essential to TOX2-SE activity. Mutations were introduced to these motifs in pGL4.26 constructs containing E1, E2, and E3 (Fig. 6G). Destroying the RUNX binding site in these TOX2-SE regions resulted in a reduction of luciferase activity from 9 -13-fold to 2—fourfold above empty vector **(**Fig. 6G). The results indicate that these binding motifs are required for optimal function of the TOX2-SE.

Collectively, our results show the SE region of TOX2 is bound by RUNX3, providing a mechanism by which SE-driven TOX2 activation is dependent, at least partially, on oncogenic transcription factor RUNX3 in NKTL.

### Confirmation of correlations among TOX2/PRL-3/RUNX3 and their prognostic values in an independent study cohort

**Fig. 7:**
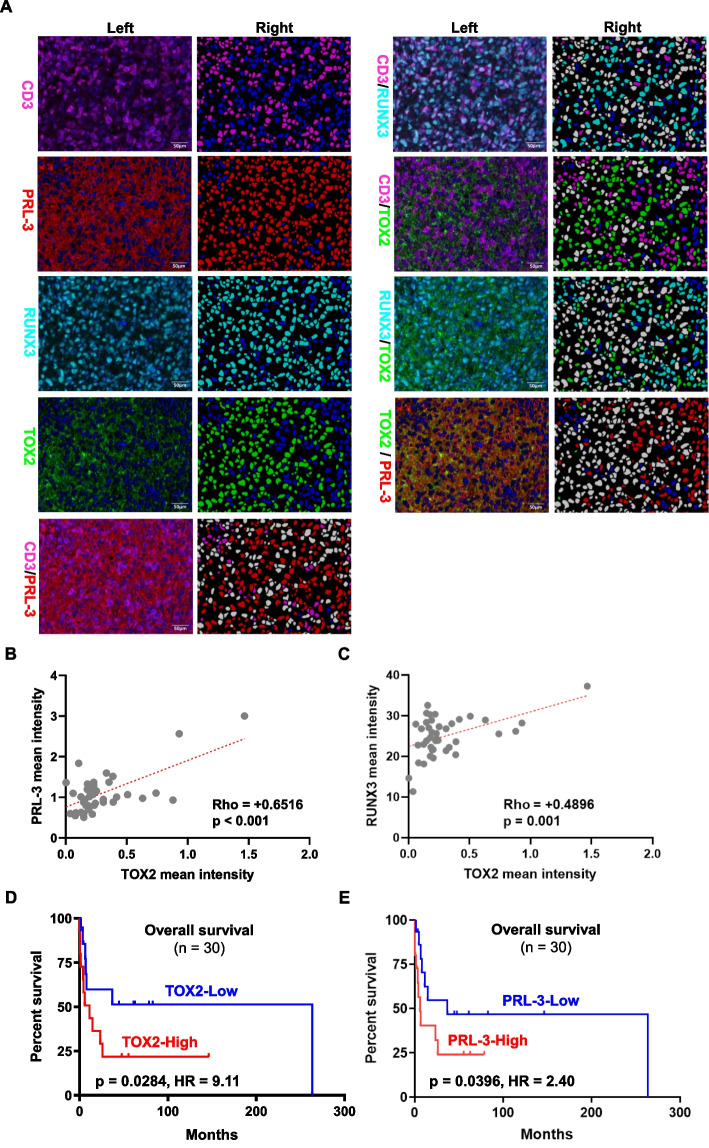
Multiplex immunofluorescence (mIF) validation of TOX2, RUNX3 and PRL-3 expression in an independent cohort of clinical samples from 42 NKTL patients (NUH). **A** Representative images of protein expression of CD3, RUNX3, TOX2 and PRL-3 in NKTL patient samples using mIF method. Left columns represented protein expression of CD3 (membrane, magenta), PRL-3 (cytoplasm, red), RUNX3 (nuclear, cyan), and TOX2 (nuclear, green) in NKTL with multiplexed immunofluorescence staining. Right columns indicated the corresponding image analysis masks. Double positive cells were in white; single positive cells were marked in the corresponding immunofluorescence staining color; while negative cells were in blue. The scale bars indicate 50 µm. **B** Correlation between TOX2 expression with PRL-3 expression in NKTL patients (*n* = 42) was determined by mean intensity of staining quantified with Visiopharm program. A significant positive correlation was determined by Pearson's *p* < 0.001, and R = 0.65. **C** Correlation between TOX2 expression with RUNX3 expression in NKTL patients (*n* = 42) was determined by mean intensity of staining quantified with Visiopharm program. A significant positive correlation was determined by Pearson's *p* = 0.001, and R = 0.50. **D** Kaplan–Meier analysis was performed on the overall survival between patients (*n* = 30) expressing higher TOX2 expression (TOX2-High, ≥ median expression) and lower (TOX2-Low, < median expression). **E** Kaplan–Meier analysis was performed on the overall survival between patients (*n* = 30) expressing higher PRL-3 expression (PRL-3-High, ≥ median expression) and lower (PRL-3-Low, < median expression). In **D** and **E** statistical significance (p) was evaluated by Log-rank test and *p* < 0.05 was considered as significant. HR: hazard ratio

To support our above-mentioned findings, we used mIF approach to further study the association between TOX2 protein expression with PRL-3 and RUNX3 in CD3 + NKTL tumor cells in an independent study cohort (supplementary Table S9). Representative images of TOX2, PRL-3 and RUNX3 expression markers in CD3 + tumors were illustrated (Fig. 7A). Our analysis showed that the mean intensity of TOX2 expression was significantly correlated with the mean intensity of PRL-3 expression (Fig. 7B**,** Pearson's R = 0.65; *p* < 0.001) and RUNX3 (Fig. 7C, Pearson's R = 0.50; *p* = 0.001). Kaplan–Meier analysis demonstrated that patients with higher TOX2 expression (TOX2-High, ≥ median expression) were associated with shorter overall survival compared to patients with lower TOX2 expression (TOX2-Low, < median expression) (*p* = 0.0284, HR = 9.11) (Fig. 7D). Similarly, patients with higher PRL-3 expression (PRL-3-High, ≥ median expression) demonstrated a worse overall survival than patients with lower PRL-3 expression (PRL-3-Low, < median expression) (p = 0.040, HR = 2.49) (Fig. 7E). Overall, our mIF data in the validation cohort confirmed the correlation of TOX2 expression with PRL-3 and RUNX3 and high levels of TOX2, PRL-3 expression were associated with poor outcome in an independent set of NKTL patient samples.

### Silencing TOX2 impairs tumorigenicity in vivo

**Fig. 8:**
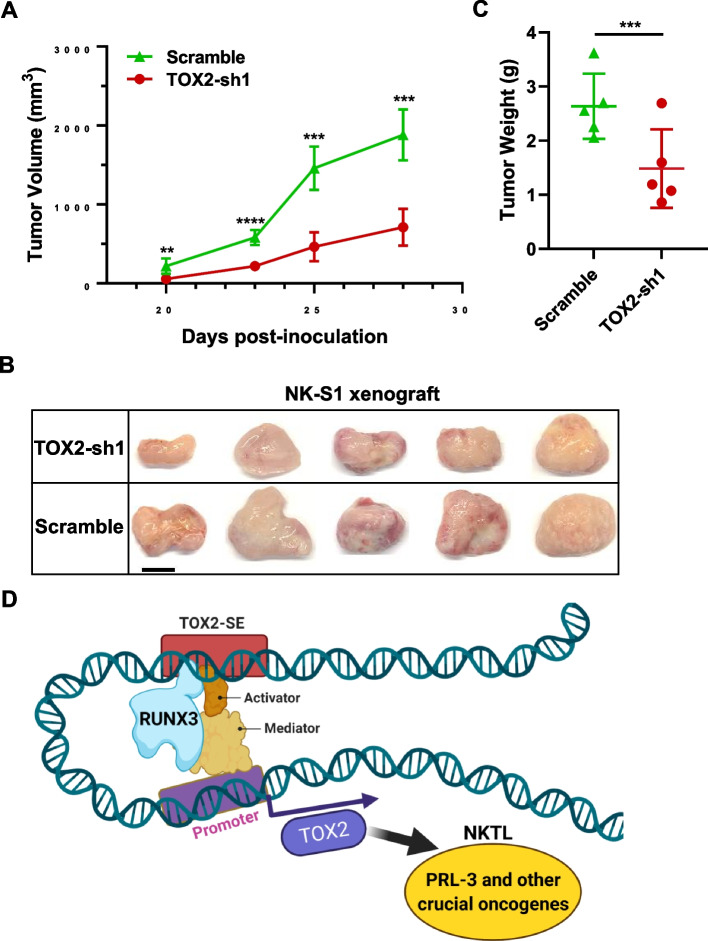
Mouse xenograft models of NK-S1-scramble and NK-S1-TOX2-sh1 cells. **A** The tumor volume was measured by caliper every 2 -3 days. The tumor growth curves were constructed according to the average tumor volume of each group ± SD (mm^3^). **B** Mice were sacrificed, and then the images of xenograft tumors were captured after dissection. Scale bar, 1 cm. **C** Tumor weights of NK-S1 xenografts in scramble (control) verse TOX2-sh1 group. *N* = 5. **p < 0.01; ***p < 0.001; ****p < 0.0001. **D** Schematic representation of molecular mechanism involving in TOX2-SE-driven oncogenesis in NKTL

To determine the tumorigenic role of TOX2 in vivo, we used this pair of NK-S1-scramble and NK-S1-TOX2-sh1 cells, to subcutaneously inject into one side of NSG mice (*n* = 5). NK-S1-scramble cells formed palpable tumor mass at day 20 post inoculation and progressed rapidly up to 1880 ± 287 mm^3^ at day 28 after cell inoculation in immunodeficient mice. Strikingly, the tumors of NK-S1-TOX2-sh1 cells developed significantly smaller in size (710.7 ± 232 mm^3^) when compared with NK-S1-scramble tumors (Fig. 8A, *p* < 0.001). The images of naked tumors were shown in Fig. 8B. Consistently, we detected a significant reduction in NK-S1-TOX2-sh1 tumor weights when compared to NK-S1-scramble tumor (Fig. 8C, *p* < 0.001). Therefore, these data suggest that TOX2 confers important oncogenic function in NKTL cells in vivo.

## Discussion

Overall, we describe the aberrant SE landscape and transcriptional program in NKTL patient samples and cell lines. To our knowledge, this is the first study providing the comprehensive changes in SE profiling and gene expression in NKTL. The analysis reveals novel insights into the pathogenesis of NKTL and uncovers TOX2 as a critical SE-associated oncogene and a potential therapeutic target.

Similar to many other types of cancer, the oncogenic transformation of NKTL cells rely on dysregulation of a core set of TFs. Consistent with this notion, we identified a list of SE-associated TFs which are important in NK/T cell functions, suggesting that SE establishment plays a key role in NKTL biology. The high mobility group box (HMG-box) superfamily are non-histone proteins, regulating DNA-dependent process by changing chromatin structure [44]. Thymocyte selection-associated HMG box (TOX), a transcription factor in HMG-box superfamily, consists of four subfamily members: TOX, TOX2, TOX3, and TOX4 [45]. Among them, the oncogenic property of TOX has been reported in T-cell acute lymphoblastic leukemia (ALL) and acute myeloid leukemia (AML) [46, 47]. In contrast, the role of TOX2 in hematological malignancies and solid tumors has not been established. TOX2 regulates the development of NK and follicular helper (Tfh) cells through TBX21 and BCL6, respectively [48, 49]. We here identified *TOX2* as a novel NKTL-SE oncogene and used this SE gene as an example for further study. We provided compelling evidence demonstrating the oncogenic function of TOX2 and dissected molecular mechanism of SEs on the activation of oncogenes in NKTL.

We found that TOX2 was not only overexpressed in NKTL primary tumors and cell lines, but also negatively associated with patient survival. We functionally characterized the impact of TOX2 gene on NKTL cell growth, cell cycle, apoptosis and colony formation. Notably, silencing TOX2 decreased the tumor size in vivo. Several downstream target genes revealed by RNA-seq analysis of TOX2-knockdown cells may account for these biological consequences. PRL-3 belongs to the phosphatase of regenerative liver (PRL) family (PRL-1, -2, -3) [50]. A number of studies from our group and others reported that PRL-3 is widely overexpressed in a majority of solid tumors and hematological malignancies [51–56]. PRL-3 has been characterized as pro-metastasis and poor prognosis factor [50, 57]. TOX2 is enriched at promoter-TSS region of *PRL-3* gene. In addition, a number of other downregulated genes play important roles in cancer cell proliferation, disease progression, poor prognosis and resistance to therapies. A large body of evidence shows that high LGALS3BP expression in tissues and serum are associated with unfavourable clinical outcomes in a wide variety of malignancies, including breast, lung, ovarian, pancreatic, prostatic, liver, gastric cancers and melanoma [58]. Adhesion to LGALS3BP has been documented as a mechanism for drug resistance in lymphoma, lung cancer and ovarian cancer [59–61]. SPP1 (Osteopontin, OPN), has diverse roles in regulation of immune response, anti-apoptosis, cellular viability, and NK cell development and function [62]. Absence of OPN in the bone marrow niche leads to a significant decreased NK population and deficiency of intracellular OPN [63]. NK cells with deficient expression of OPN display defective responses to IL-15 and diminished responses to metastatic tumors [64]. OPN has been widely implicated in cancer invasion and metastasis, poor prognosis and resistance to radiation and chemotherapy through promoting cancer stem cell-like properties and binding with CD44 or integrin receptors [65–67]. Other important genes in this list downregulated by TOX2-shRNA, such as *ITGB7* [68], *SLAMF1* [38], *CD244* [69], *DPYSL3* [70], *KRT80* and *KRT7* [71, 72], have been implicated in cancer progression or drug resistance.

Taken together, the list of genes affected by TOX2 elimination play pivotal roles in drug resistance, cancer progression, metastasis, and worsen clinical outcomes. Collectively, they drive the development of NKTL and contribute to therapy resistance and disease progression in patients with NKTL.

Using the CRISPR/dCas9 interference tool, sgRNAs targeting five different constituent sites on the SEs of *TOX2* significantly reduced the TOX2 transcription and protein levels. Consequently, we observed attenuated cell proliferation and induced cell apoptosis in NKTL cells. Our findings suggested *TOX2* as a novel SE-controlled oncogene in human NKTL. Importantly, we uncovered a positive correlation between TOX2 and RUNX3 mRNA and protein expression in NKTL patients. EBV contributes to several types of human cancers, including NKTL. Earlier studies demonstrated that the EBV infection induces RUNX3 expression regulated by EBNA2 [42, 73]. Until recently, Zhou and colleagues delineated the landscape of EBV-activated super-enhancers (EBV-SEs) for the first time [74]. Interestingly, EBNA2-SEs are found to be localized near RUNX3 genes in EBV-transformed lymphoblastoid cell lines (LCLs) [74]. Subsequently, two independent studies confirmed that SEs for RUNX3 are required for cell proliferation in EBV-infected B cells [75, 76]. In this study, we demonstrate, for the first time, that RUNX3 binds to TOX2-SE and drives TOX2 expression NKTL tumors. NKTL is an EBV-associated cancer. In addition, consensus sequences of RUNX3 is enriched at the binding sites in the SE region of RCAN1.4 in breast cancer [77]. Based on these findings in EBV-infected B cells, it therefore suggests that EBNA2 might induce RUNX3 expression in EBV-infected NKTL cells. Consequently, overexpressed RUNX3 increases TOX2 transcription through the binding to TOX2-SE. Furthermore, among these 65 genes for their genomic regions overlapping with TOX2 binding (Supplemental Table S6), two potential TOX2 binding sites were identified in the intron 3 of *TOX2* gene (NM_001098797). These data imply that TOX2 could create a positive regulatory feedback loop that establishes expression of TOX2 in NKTL.

RUNX3 has been implicated as a tumor suppressor or oncogene in different type of cancers [78]. We and others demonstrated that RUNX3 is oncogenic and its overexpression is correlated with poor prognosis and drug resistance in NKTL and anaplastic large cell lymphoma (ALCL) [17, 79, 80]. Interestingly, RUNX3 expression is also regulated by super enhancers in EBV-positive malignant B cells [76]. In this study, RUNX3 is recruited and bound to the SE of *TOX2*, thus further driving expression of the SE-related oncogene in cooperation with mediator complex and other TF co-activators. The RUNX3-TOX2-SE-TOX2-PRL-3 regulatory pathway may represent a hallmark of NKTL biology, which could be therapeutically exploited (Fig. 8D). BET (bromodomain and extra-terminal domain) proteins and cyclin-dependent kinases (CDKs) are key components of super-enhancer. A number of BET inhibitors and CDK inhibitors have being tested in different phases of clinical trials in hematologic malignancies [30]. Our study provides rational for evaluating the clinical efficacy of these inhibitors in NKTL patients. Although, direct TOX2 inhibitor is not available, developing proteolysis-targeting chimera (PROTAC) molecules targeting TOX2 is a promising strategy, supported by the fact that two PROTAC degraders (ARV-110 and ARV-471) have progressed into phase II clinical trials [81]. PRL3-zumab, a First-in-Class humanized antibody drug against PRL-3 oncoprotein, has been approved for Phase 2 clinical trials in Singapore, US, and China to treat all solid tumors [82, 83]. Therefore, examining the clinical utility of PRL3-zumab against NKTL is timely needed.

In conclusion, we, for the first time, describe the SE landscape discovery in NKTL cells. We use TOX2-SE as example to demonstrate that the discovery strategy of key SE-associated genes in the current study is a useful tool for uncovering novel, cancer-unique oncogenes. The changes in SE-dependent regulatory networks such as RUNX3-TOX2-SE-TOX2-PRL-3 identified in this study offer valuable opportunities for therapeutic targeting NKTL.

## Supplementary Information

**Additional file 1: Supplemental Table S1.** Characteristics of NKTL cell lines and culture conditions. **Supplemental Table S2.** The clinicopathological characteristics of these 3 NKTL patients.** Supplemental Table S3.** The list of primers and shRNA and their sequences.** Supplemental Table S4.** The list of SE-genes, RNA-seq overexpression genes and their common genes.** Supplemental Table S5.** shRNA screening identifies shared downregulated, upregulated genes affected by TOX2-shRNA1 and TOX2-shRNA2 in NKYS cell.** Supplemental Table S6.** TOX2 binding on 12 genes out of 65 genes downregulated by TOX-shRNA.** Supplemental Table S7.** Identification of TOX2 binding motif (^VSSSGVVGCG) in PTP4A3 promoter.** Supplemental Table S8.** RUNX3 binding motifs on TOX2-SE and their ChIP-PCR primers.** Supplemental Table S9.** Clinical features of 42 cases of NKTL and their expression of TOX2, RUNX3 and PRL-3 in CD3+ NKTL tumor cells.** Figure S1. **Determination of cell-origin of 3 NKTL cases on their RNA-seq data. This analysis was performed by using a two-sample Kolmogorov-Smirnov based method developed in house and the NK cell and T cell signature published by Xiong J, et al (Cancer Cell. 2020 Mar 16;37(3):403-419). This dot plot shows the genes expression of NK-origin (blue) and T-cells-origin (red). Lowly expressed genes (mean FPKM < 1) has been filtered out.** Figure S2.** UCSC Genome Browser ChIP-Seq screenshot. Track view of H3K27ac ChIP-seq density profile centered at the TBX21 (T-bet) and EOMES gene loci of NKTL cell line HNAK1 and NKYS (top panel), 3 tonsil controls (middle panel) and 3 primary NKTL patient samples (lower panel). Locations of the SEs regions were marked by red bars.** Figure S3. **Gene network of NKYS cells responding to TOX2 knockdown derived from GeneMANIA. A gene network from GeneMANIA shows the relationships for genes from the list of downregulated genes induced by TOX2-shRNAs according to the functional association networks from the databases. TOX2 sat on the top of the network and a physical interaction of network, indicated by a red arrow. Black rectangles highlight a few important targets, including PTP4A3, SPP1, SLAMF1, CD244, ITGB7. A black circle comprises some MHC family members.** Figure S4.** The expression of RUNX1 and RUNX3 in normal NK cells, NKTL cell lines and NKTL patient samples. Expression (lg2) level of RUNX1 and RUNX3 in a collection of normal NK cells, NKTL cell line and NKTL patient samples derived from a microarray dataset in Gene Expression Omnibus (GEO) database (accession number: GSE80632). p value < 0.05 is considered as statistically significant. **Figure S5.** TOX2 overexpression rescues NKYS cells from RUNX3 depletion. (A) Comparison of the rate of growth of co-transduced NKYS cells expressing FLAG-EV (empty vector) or FLAG-TOX2 and RUNX3-sh1 constructs. NKYS cells co-transduced with FLAG-EV and scramble shRNA were used as control. For each condition, cell number was counted at day 2, 4, and 6, then converted to fold change relative to the starting number at day 0. Same number of cells were seeded at day 0 and comparison was made at indicated days for relative fold changes. Three biologically independent experiments were performed (mean ± SD). Western blot analysis showing the expression of RUNX3 and TOX2 shown on the right. β-actin was used as the loading control.

## Data Availability

The datasets supporting the conclusions of this article are available in the GEO repository. All H3K27ac ChIP-Seq data were deposited in the GEO database (Accession number: GSE190925). All RNA-Seq data were deposited in the GEO database (Accession number: GSE189632).

## Abbreviations

SE: Super enhancer
NKTL: Natural killer/T cell lymphoma
ALCL: Anaplastic large cell lymphoma;
EBV: Epstein-Barr virus
ALL: Acute lymphoblastic leukemia
AML: Acute myeloid leukemia
TSS: Transcription start site
TF: Transcriptional factor
TOX: Thymocyte selection-associated HMG box
HMG-box: High mobility group box
PRL-3: Phosphatase of regenerative liver 3
ChIP: Chromatin (Ch) immunoprecipitation (IP)
shRNA: Short hairpin RNA
CRISPR: Clustered Regularly Interspaced Short Palindromic Repeats
Cas9: CRISPR-associated protein 9
dCas9: Cas9 endonuclease dead
sgRNA: Single guide RNA
GFP: Green fluorescent protein
