# Supplementary material for: Super-enhancer-driven TOX2 mediates oncogenesis in Natural Killer/T Cell Lymphoma
Source: Mol Cancer. 2023 Apr 10;22:69. doi: 10.1186/s12943-023-01767-1 (PMC10084643; doi:10.1186/s12943-023-01767-1)
Supplement: Supplementary file 1 — Additional file 1: Supplemental Table S1. Characteristics of NKTL cell lines and culture conditions. Supplemental Table S2. The clinicopathological characteristics of these 3 NKTL patients. Supplemental Table S3. The list of primers and shRNA and their sequences. Supplemental Table S4. The list of SE-genes, RNA-seq overexpression genes and their common genes. Supplemental Table S5. shRNA screening identifies shared downregulated, upregulated genes affected by TOX2-shRNA1 and TOX2-shRNA2 in NKYS cell. Supplemental Table S6. TOX2 binding on 12 genes out of 65 genes downregulated by TOX-shRNA. Supplemental Table S7. Identification of TOX2 binding motif (^VSSSGVVGCG) in PTP4A3 promoter. Supplemental Table S8. RUNX3 binding motifs on TOX2-SE and their ChIP-PCR primers. Supplemental Table S9. Clinical features of 42 cases of NKTL and their expression of TOX2, RUNX3 and PRL-3 in CD3+ NKTL tumor cells. Figure S1. Determination of cell-origin of 3 NKTL cases on their RNA-seq data. This analysis was performed by using a two-sample Kolmogorov-Smirnov based method developed in house and the NK cell and T cell signature published by Xiong J, et al (Cancer Cell. 2020 Mar 16;37(3):403-419). This dot plot shows the genes expression of NK-origin (blue) and T-cells-origin (red). Lowly expressed genes (mean FPKM < 1) has been filtered out. Figure S2. UCSC Genome Browser ChIP-Seq screenshot. Track view of H3K27ac ChIP-seq density profile centered at the TBX21 (T-bet) and EOMES gene loci of NKTL cell line HNAK1 and NKYS (top panel), 3 tonsil controls (middle panel) and 3 primary NKTL patient samples (lower panel). Locations of the SEs regions were marked by red bars. Figure S3. Gene network of NKYS cells responding to TOX2 knockdown derived from GeneMANIA. A gene network from GeneMANIA shows the relationships for genes from the list of downregulated genes induced by TOX2-shRNAs according to the functional association networks from the databases. TOX2 sat on the top of the network and a [file 12943_2023_1767_MOESM1_ESM.pdf]

SUPPLEMENTAL INFORMATION

Super-enhancer-driven TOX2 mediates oncogenesis in Natural Killer/T Cell Lymphoma

Supplemental Methods and Materials

RNA extraction and real-time quantitative PCR analysis

Total RNA was extracted using the miRNeasy Mini Kit (Qiagen GmbH, Germany) protocol with the DNaseI treatment included. A reverse transcription (RT) reaction was carried out by using the High Capacity cDNA Reverse Transcription Kit system (Thermo Fisher Scientific, MA, USA). Real-time fluorescence monitoring of the PCR products was assayed with SYBR green (Thermo Fisher Scientific) with the 7500 Fast Real-Time PCR (RT- PCR) System (Thermo Fisher Scientific). Relative gene expression levels were calculated using $2^{(-\Delta\Delta CT)}$ by comparing the amount of endogenous *GAPDH* in the same sample. All the primer details were listed in supplemental Table S3.

Lentiviral infection

Lenitviral supernatants were produced by co-transfection of scramble shRNA or TOX2-shRNAs or RUNX3-shRNAs together with packaging plasmids into 293T cells. Three million of HANK1 or NKYS cells were mixed with concentrated viral supernatant and 8 µg/ml of polybrene (Millipore) and centrifuged at 2500 rpm for 90 min at 30ºC. After additional incubation at 37ºC for 4 hours, the medium was changed to fresh complete medium. Three days later, cells were submitted for RNA and protein extraction, followed by qRT-PCR and Western blot analysis of TOX2 or RUNX3. Primers were listed in supplemental Table S3.

RNA-seq data processing

The RNA-seq data were checked for raw sequence quality using FastQC v0.11.5, and were filtered to remove adaptor sequences, contamination and low quality read. The sequences were mapped to human genome hg38 using STAR v2.4.2a and subsequently, transcript quantification using RSEM 1.2.25 with Gencode v24 annotation. EBseq v1.20.0 was used to identify differentially expressed genes. T-test was computed by Matlab® R2012a, statistics toolbox version 8.0 (MathWorks; Natick, MA).

Chromatin immunoprecipitation (ChIP)-PCR

ChIP was performed using SimpleChIP® kit according to the manufacturer's instruction (Cell Signalling Technology, USA). Briefly, NKYS and HANK1 cells were crosslinked in 1%

formaldehyde for 10 minutes. The cross-linking reaction was stopped by the addition of Glycine for 5 minutes. After washing and lysis, samples were digested using micrococcal nuclease and sonicated on ice to an average size of 150 - 900 bp and centrifuged for 10 min at 10 000 *g* at 4°C to remove insoluble material. 2% of this solution was saved (input chromatin) and processed with the eluted immunoprecipitates beginning with the cross-link reversal step. Samples were incubated overnight at 4°C with anti-RUNX3 antibody (sc-376591 X, Santa Cruz Biotechnology, TX, USA) or isotype matched control IgG (negative control) with rotation. Immune complexes were collected by incubating samples with protein G magnetic beads for 2 hrs at 4°C with rotation. The beads were pelleted by brief centrifugation and washed as per the manufacturer's instructions. The immunocomplexes were eluted from the magnetic beads by elution buffer. Input and immunoprecipitated chromatin were incubated with elution buffer at 65°C for 2 hours to reverse DNA–protein cross-links. The immunoprecipitated DNA was purified as per the manufacturer's instructions (Merck, Millipore, USA) and analysed by qPCR. Fold enrichment method was used to calculate the increase in specific signal (anti-RUNX3 antibody) over back ground noise (IgG control antibody). The values were then normalized to the values of IgG control, which was defined as 1. The ChIP-PCR primers amplifying specific TOX2-SE regions containing RUNX3 binding motifs were included in supplemental Table S6.

Supplemental tables

Supplemental Table S1. Characteristics of NKTL cell lines and culture conditions.

Cell lines	Sex	Age	Ethnicity	Original description of disease	CD56	EBV	Cytokine dependency	Culture conditions
NK-92	M	50	Caucasian	ANKL	+	+	IL-2	RPMI-1640 medium, 10% FBS
NK-S1	M	58	Chinese	NKTL	-	+	-	DMEM medium, 10% FBS, 10% equine serum
NK-YS	F	19	Japanese	NKTL	+	+	IL-2	RPMI-1640 medium, 10% FBS
HANK-1	F	46	Japanese	NKTL	+	+	IL-2	Artemis medium-2, 2% human serum

ANKL, aggressive NK-cell leukemia FBS: fetal bovine serum

Supplemental Table S2. The clinicopathological characteristics of these 3 NKTL patients.

UPN	Sex	Age	Race	Ann Arbor stage	IHC markers for diagnosis	EBV DNA copies/ml plasma
NKTL9	M	25	Emirati	IV	CD30+, CD2+, CD3+, TiA1+, GranzymeB+, EBER+, CD56+, Ki67 90-100%, CD10+, CD20+, CD21+, CD5-, CD8-, TDT-, CD4-	288
NKTL10	M	56	Chinese	IV	CD2+, CD43+, CD8+, TiA1+, EBER+, TCRbeta+, Ki67 ~40%, CD3-, CD5-, CD7-, CD4-, CD30-, TCRgamma-, CD56-, ALKinase-, CD20-, PAX5-, CD79a-	127, 160
NKTL4	M	51	Chinese	IIIE	CD3+ CD56+, GranzymeB+, EBER+, CD138+, Ki67 <10%, TiA1+ CD2+, CD4+, Lyn+, Lck+, TDT-, CD5-,CD20-, CD8-, CD7-	N.A.

Supplemental Table S3. The list of primers and shRNA and their sequences.

**Name**	**Sequences**
TOX2-shRNA1	AGCAAAGAAGGAATATCTGAA
TOX2-shRNA2	ACTTTCGGTGACGTGTCCAAA
RUNX3-shRNA1	GGCTAGCAGCATGCGGTATTT
RUNX3-shRNA1	ACCTCGGAACTGAACCCATTC
**qRT-PCR**	
TOX2-Forward	5'-CTCAGGAAGAGGAGTCGGAAGT-3'
TOX2-Reverse	5'-ACACAGGCTTCTGCGGCTCATT-3'
RUNX3-Forward	5'-ATGACGAGAACTACTCCG-3'
RUNX3-Reverse	5'-TCAGGGTGAAACTCTTCC-3'
EOMES-Forward	5'-AAATGGGTGACCTGTGGCAAAGC-3'
EOMES-Reverse	5'-CTCCTGTCTCATCCAGTGGGAA-3'
TBX21-Forward	5'-ATTGCCGTGACTGCCTACCAGA-3'
TBX21-Reverse	5'-GGAATTGACAGTTGGGTCCAGG-3'
ID2-Forward	5'-GGCGAGTGCGGATAAAAGC-3'
ID2-Reverse	5'-CTAACGGACCTCACGGGACT-3'
CD150-Forward	5'-AGGCCCTCCACGTTATCTA-3'
CD150-Reverse	5'-GCAAAAGCGCTGAACTGA-3'
CD244-Forward	5'-CTGCTTCTGTGTGTGGAGGA-3'
CD244-Reverse	5'-AGCAGAAGACTGGGACTGGA-3'
ITGB7-Forward	5'-GAATCAACCAGACGGTGACTTTCT-3'
ITGB7-Reverse	5'-GCCCGGAGCCTCAGGA-3'
GAPDH-Forward	5'-GTATTGGGCGCCTGGTCAC-3'
GAPHD-Reverse	5'-CTCCTGGAAGATGGTGATGG-3'
**PCR for TOX2-SE**	KpnI sequence: GGTACC; NheI sequence: GCTAGC
TOX2-eNC-F	5'-TTAGCAGGTACCGGATAACCCAGAACATAAAAC-3'
TOX2-eNC-R	5'-TGCTTAGCTAGCACCTCAATGAACACTGGTTTGC-3'
TOX2-e1-F	5'-TTAGCAGGTACC ATTCGTATAGTCAGACATGAGT-3'
TOX2-e1-R	5'-TGCTTAGCTAGCCCATTTAAAATCCTTGGACGGCC-3'
TOX2-e2-F	5'– TTAGCAGGTACCGCAGGATGGATGCTCAGAAGTC-3'
TOX2-e2-R	5' – TGCTTAGCTAGCATGAATGAATGAATGATCCTGAGC-3'
TOX2-e3-F	5'–TTAGCAGGTACCAATCAGTCCTAGCACTTTGGGAG-3'
TOX2-e3-R	5'– TGCTTAGCTAGCCTAAAGCTCACGCTTATAACAGC-3'

**Mutant RUNX3 binding site**	**Site-direct mutagenesis primer sequences**
MUT-RUNX3-E1	5'-CATATCTGAGCATATCCC**TAACGTCA**CTCAGCAGCAGCACC-3'<br>5'-GGTGCTGCTGCTGAG**TGACGTTA**GGGATATGCTCAGATATG-3'
MUT-RUNX3-E2	5'- CGGCCTTATCTGATGC**TAACGTTG**GGGAGCCAGCGAG-3'<br>5'- CTCGCTGGCTCCC**CAACGTTA**GCATCAGATAAGGCCG-3'
MUT-RUNX3-E3	5'-CGTCGGGTTGCACTG**TAACGTGG**GGGGAACGAGTG-3'<br>5'-CACTCGTTCCCC**CCACGTTA**CAGTGCAACCCGACG-3'
**TOX2 ChIP-PCR**	**Primers sequences**
P1 (-188 to +33)	Forward: 5’-GCACAGGAGCCAAGGTCAGT-3’<br>Reverse: 5’-TGGAGGGCTCGGGACGCGCG-3’
P2 (+14 to +168)	Forward: 5’-TCGTGCCGGCGCCGCCCGGA-3’<br>Reverse: 5’-AGCCGTGCCCGGAGCGGGC-3’
P3 (-864 to -629)	Forward: 5’-GGTGGATGGGTGGAAAAGCA-3’<br>Reverse: 5’-CTTATGGGTCAGCCGTTCCT-3’

Supplemental Table S4. **The list of SE-genes, RNA-seq overexpression genes and their common genes.**

SE-associated genes	Overexpression genes by RNA-seq	Common genes
EOMES	BCORP1	ITPK1
RUNX2	NLGN4Y	SIGLEC9
TBX21	RPS4Y1	SLC8A1
ADSS	EIF1AY	CST7
ANKRD28	KDM5D	WNT8B
ANTXR2	TXLNG2P	DUSP4
APMAP	UTY	RBPJ
ARHGAP18	DDX3Y	DNMT3B
ARHGAP30	PRKY	EXOC2
ARID1A	ZFY	RGL1
ARRB1	USP9Y	PHLDA1
AUTS2	TTTY15	NCAM1
BCAT1	KLRC1	GPR171
C15orf39	MT-RNR2	HIST1H2BJ
C19orf54	RP4-742C19.12	SPRY2
CADM1	KIR2DL4	KIF14
CCL5	KRT81	GFI1
CD3E	GZMAP1	SH2D2A
CDC7	TRDJ1	KLRC1
CENPF	MT-RNR1	MAGOHB
CEP55	CCL13	TBX21
CEP57L1	NCAM1	RASSF4
CERS6	CTSW	MTHFD1
CHN2	NMUR1	PXN
CHSY1	GDF6	ATP8B4
CKAP2L	SLC4A10	BVES
CLDND2	CCL3	RAB20
CMC1	KLRC2	CHN2
COLGALT2	KRT86	FCGR3A
COMMD7	APOBEC3H	EAF1
CRIM1	UBBP4	PRF1

CST7	XCL2	CMKLR1
CTSC	TRDC	PDE4D
CXCR3	RP11-291B21.2	YPEL1
DENND1B	SH2D1B	RASGEF1A
DFFA	ITGAD	SLC19A1
DKC1	CD163	EOMES
DLGAP5	KLRD1	SLC5A3
DMXL2	NCR1	HIST1H2AG
DOK2	RNF157	TPST2
DPP4	AC021218.2	STK39
DPYSL2	GZMB	KIF23
DRAM1	FCGR1B	GNLY
DUSP4	MRC1	COL4A2
EAF1	GZMA	GRAMD1B
ECI2	FCGR3A	GATA3
ETFB	CXCL9	PTGER2
EXOC2	RP4-738P11.4	ITGAD
F2R	MSR1	DMXL2
FAM102B	SLC22A20	MS4A4A
FASLG	FASLG	SNX18
FCGR3A	MLC1	CXCR3
FHL3	KLRC3	TMPRSS3
GAS7	SLC27A2	CEP55
GATA3	NKG7	F2R
GFI1	MATK	GZMB
GLUL	RP4-738P11.3	C15orf39
GPR65	SATB2	NCL
GSK3A	HIST3H2BA	ID2
GYG1	PLCH1	TOX2
GZMA	PTGER2	SH2D1B
GZMK	RP11-121A8.1	RUNX2
HENMT1	JAKMIP1	DLGAP5
HIST1H2AG	PTGDR	MAFB
HIST1H2AL	XCL1	GAB3
HIST1H2BJ	LILRB5	CKAP2L

HIST1H2BO	DDN	CCL5
HIST1H3G	PRF1	YARS
HIST1H3I	FN1	TGFBR3
HJURP	TRGV9	S100PBP
HLTF	RP11-120K18.2	ABCA2
ID2	SIGLEC1	DOK2
ITGA1	HK3	HELZ E
ITPK1	SMKR1	ETFB
KIF14	KLRC4-KLRK1	SLC27A2
KLRC1	DPP4	ADSS
KLRD1	GZMH	SNTB1
MAGOHB	PI15	CERS6
MATK	MRC1L1	GYG1
MICAL2	C1QB	SUCNR1
MSL3P1	KLRC4	B4GALT5
MTBP	MTRNR2L12	HIST1H2BO
MTHFD1	CCL4	AUTS2
MTHFD2	SPAG6	POMGNT2
MYO1F	GNLY	HAVCR2
NCALD	HPDL	CD3E
NCAM1	DNMT3B	ARID1A
NCL	CD244	PDE4A
NKG7	RP11-484L8.1	ZADH2
PDE4A	FAM172BP	CCL13
PDE4D	OASL	ZAP70
PHLDA1	LILRA3	PLAU
PRDM8	SLC4A4	MATK
PRF1	AC010970.2	HLTF
PRR5L	CATSPER1	KCNE1
PTGDR	SERPINE1	FHL3
PTGER2	CXCL11	DPYSL2
PTP4A1	AC069363.1	SH2D1A
RAD51D	GZMK	SAMD3
RASGEF1A	HIST3H2A	CHSY1
RASSF4	EOMES	ARHGAP30

RBPJ	LILRA6	RGS3
RGL1	FAM47E-STBD1	PTGDR
RGS3	LGALS1	GDF6
RNF125	HIST3H2BB	DPP4
RNF213	DHRS2	HIST1H3I
RPS6KA3	KIR3DX1	HENMT1
RRP12	CELSR3	TIPARP
S100PBP	C4orf50	FAM102B
SAMD3	AC104135.3	SEC31B
SATB2	PPP2R2B	ANKRD28
SCD	CCDC74A	ZBTB2
SEC31B	FCER1G	TSEN15
SH2D1A	COLGALT2	COMMD7
SH2D2A	TYROBP	SLC25A44
SLC16A6	GBP5	APMAP
SLC19A1	APOL4	ZNF609
SLC20A1	KLHL4	ITGA1
SLC25A44	SPRY2	COLGALT2
SLC27A2	FOXD2-AS1	HIST1H2AL
SLC4A10	MT2A	ANTXR2
SLC5A3	C1QA	GZMK
SLC8A1	HSD11B1	MS4A6A
SLX4IP	FAM179A	NKG7
SMYD2	LILRB2	MTHFD2
SNTB1	SAMD3	SATB2
SNX18	PLTP	JAKMIP1
SPRY2	SERPING1	ZYX
SUPT3H	RP11-214O1.2	SUPT3H
TESC	SLC6A12	SCD
TGFBR3	CST7	CMC1
TIPARP	TIMP1	GALC
TNFSF14	LINC00484	DRAM1
TOX2	C19orf57	CRIM1
TPST2	FCGR2A	HIST1H3G
TSEN15	AMH	ARRB1

UBASH3A	MANEAL	DAPK2
YARS	F2R	CTSD
YPEL1	TSPAN2	DKC1
ZADH2	CTD-2377D24.6	MTBP
ZAP70	TRGV10	SMYD2
ZBTB2	MT-ATP8	ECI2
ZNF367	SLAMF8	CDC7
ZYX	CCL5	MVB12B
ABCA2	USP44	UBASH3A
ATP8B4	CCL18	SLC4A10
B4GALT5	RP11-539L10.2	RNF213
BVES	C1QC	CEP57L1
CCL13	AC015849.16	FASLG
CMKLR1	MS4A4A	NMUR1
COL4A2	FAM26F	PRDM8
COL6A3	TCN2	GZMH
CTSD	TRGV8	GPR65
DAPK2	TESC	DFFA
DHRS2	S100A4	ARHGAP18
DNMT3B	NRROS	MSL3P1
ENC1	CCR1	SLC16A6
GAB3	RP11-357H14.17	GZMA
GALC	EDA2R	DENND1B
GDF6	LDHAP4	IFNG
GNLY	DTHD1	GLUL
GPR171	C1orf61	SLC20A1
GRAMD1B	WNT8B	MICAL2
GZMB	F13A1	ZNF367
GZMH	CXCR2P1	PRR5L
HAVCR2	BATF2	SLX4IP
HELZ	SCD	C19orf54
HSD11B1	GCNT1	LINC00707
IFNG	ITPRIPL1	RRP12
ITGAD	SH2D2A	CADM1
JAKMIP1	GALNT15	HJURP

KCNE1	CD160	NCALD
KIF23	CXCL10	GAS7
LINC00707	IFNG	RAD51D
MAFB	CLDND2	MYO1F
MS4A4A	TIGD4	RNF125
MS4A6A	GBP1	COL6A3
MVB12B	IL2RB	RNF130
NMUR1	KCNE1	ENC1
PLAU	IL32	PTP4A1
POMGNT2	CTD-2313F11.1	CLDND2
PXN	SCARNA22	HSD11B1
RAB20	RP11-222K16.2	GSK3A
RNF130	IL10	BCAT1
SH2D1B	RP11-290F20.3	TESC
SIGLEC9	DYSF	TNFSF14
STK39	FCN1	KLRD1
SUCNR1	SEC31B	CENPF
TMPRSS3	SULF1	RPS6KA3
WNT8B	FAH	DHRS2
ZNF609	GREB1	CTSC
ERF	CD209	
ACSF3	PILRA	
ACTR1B	FCRL6	
ACVR2A	CLEC12A	
ADA	SH2D1A	
ADA2	CRISPLD2	
ADAMTS6	MVB12B	
AFAP1L2	HENMT1	
AGFG2	FOXD2	
AKAP5	FPR2	
ANKMY1	FAM64A	
ANKRD11	TNFSF14	
AP1G2	TNFRSF8	
AP5S1	RP11-277P12.20	
ARMC9	BSN	

ARNT	TBX21	
ASAH2B	TOX2	
ASB7	ACE	
ASXL2	IFITM2	
ATG7	CLEC5A	
AZI2	LRRC37A9P	
B3GNT2	IL12RB2	
B3GNT7	GIMAP4	
BAK1	C2	
BANP	SNORA71C	
BCAS2	PYHIN1	
BCL2L11	PSTPIP2	
BCYRN1	SDC3	
BLMH	RNU5E-1	
BLOC1S2	KIF14	
BLOC1S6	TMEM150B	
BNC2	ZMYND10	
BTBD3	SLFN13	
C17orf67	TMPRSS3	
C3orf58	RDM1	
C3orf67	RAET1K	
C5	AP000783.1	
C9orf139	SNORA84	
CARD19	FGR	
CATSPERE	CENPF	
CCDC107	SPOCK1	
CCDC186	SKA3	
CD200R1	LANCL3	
CDC37	CHN2	
CDK1	SLC38A5	
CDKN1A	IFITM1	
CDKN2B-AS1	SLC39A14	
CENPS-CORT	ABCA17P	
CHCHD5	SIGLEC17P	
CHD3	COL4A1	

CIC	AC017002.2	
CNIH3	LINC00707	
CNIH4	ATP8B4	
COMMD1	BVES	
COMMD6	CADM1	
COMMD9	CD3E	
CPD	HSPA6	
CSGALNACT2	CMTM1	
CSTF1	HCST	
CWC27	CKLF	
DAXX	FOXM1	
DDX1	POLR3G	
DDX12P	C12orf75	
DGKE	RHOU	
DNAJB14	HLTF	
DNAJB6	GYG1	
DNAJC8	PSME2P2	
DRAXIN	HIST1H2BO	
DUSP28	LINC00888	
DYNLRB2	STK39	
DZIP3	C7orf13	
EDEM3	CCR5	
EEF1AKMT2	ABCA2	
EFNA5	MARCO	
EIF1AX	KLRK1	
EIF3D	HOXB4	
EIF4G2	ANKRD18B	
ELL	KIF15	
ELL2	SYCE2	
ENKUR	RUNX2	
ERCC6L2	LILRA5	
ERMP1	PHLDA1	
EXOSC7	SPN	
EXT1	SUCNR1	
EXTL2	ATP10A	

FAM129A	PLCB1	
FAM133B	WNT10B	
FAM192A	ADAMTS17	
FBXL17	CDIP1	
FBXW8	RP5-857K21.11	
FCGR3B	HOXB7	
FHIT	DAPK2	
FKBP1A	ANKRD13B	
FKBP1A-SDCBP2	AUTS2	
FLJ42351	PLEKHG4	
FLOT1	SAPCD2	
FOCAD	ITGA1	
FOSB	RP11-1094M14.8	
FOXB1	STXBP5L	
FSIP1	APOBR	
FUT7	TGFBR3	
GABARAPL1	MYO1F	
GATA3-AS1	MICAL2	
GNPTAB	HIST1H2BE	
GOLGA4	KIF18B	
GPR176	PLA2G16	
GTDC1	AC093673.5	
GTPBP8	MCM10	
H2AFZ	PDF	
HERC1	RP11-473M20.7	
HIF1AN	HAVCR2	
HIKESHI	HLA-G	
HIST1H1B	STOX1	
HIST1H2AM	TLR8	
HIST1H2BH	CLSPN	
HIST1H2BI	DTL	
HIST1H3A	CRTAM	
HIST1H3J	SNORA80B	
HIST1H4A	DNAJC6	
HIST1H4B	SNORA80	

HIST1H4H	PSAT1	
HIST1H4L	SSBP4	
HLA-F	SFMBT2	
HLA-F-AS1	GOLM1	
HOPX	CRIM1	
ID2-AS1	RP1-90J20.12	
IFT52	KCNJ10	
IGF2R	KIAA1161	
IMMP2L	RP11-452D12.1	
ISCA1	RORB	
KCMF1	XRCC2	
KIAA0319L	ARHGAP18	
KIAA0513	ANKRD30BL	
KPNB1	FHL3	
L3MBTL3	IFI6	
LAT	COL6A3	
LIMD1	RP4-647J21.1	
LIMD1-AS1	ID2	
LINC00271	RP11-259N19.1	
LINC00471	TRIM46	
LINC00886	RP11-1094M14.5	
LINS1	HIST1H2BJ	
LMO4	PRDM8	
LOC100128242	POSTN	
LOC100507506	NT5M	
LOC100507507	GFOD1	
LOC101926948	MKI67	
LOC101928847	CCL2	
LOC101929541	RNF125	
LOC101929710	RNU5D-1	
LOC102724084	ADCY3	
LOC102724814	PDP2	
LOC105370941	HTATSF1P2	
LOC152048	GZMM	
LOC256880	APOBEC3G	

LOC642846	MIR663A	
LOC729291	CTSL	
LRCH4	IL2RA	
LRP2	TEX30	
MAGEF1	TMEM229B	
MAN1A1	TMEM63C	
MAP3K7CL	HIST1H2AB	
MAP4K1	PRSS1	
MAPK1	ADORA3	
MCM5	ADAMTS4	
METTL18	PRR11	
MFSD1	IQGAP2	
MIIP	IPCEF1	
MIR1181	CCDC15	
MIR6125	GOLIM4	
MKKS	RP11-1094M14.4	
MLLT3	GATA3	
MMAA	GRAMD1B	
MOB1A	FRMPD3	
MRPL13	SNORA34	
MRPL44	TLN2	
MRPS6	HIST1H3I	
MYOF	SERPINA1	
MZT1	PRAF2	
NAA38	TMEM97	
NAP1L1	LCP2	
NCOA2	TSEN15	
NDUFA9	ZNF74	
NDUFAF2	SKA1	
NDUFB8	HIPK2	
NECTIN3	PDE4A	
NEK2	TLE6	
NEPRO	DDX11L2	
NFIA	CTSB	
NGRN	SLC39A8	

NMRK1	MSL3P1	
NOL11	HIST1H2AL	
NOP10	SIGLEC9	
NTRK1	HIST1H3G	
NUDT15	SNX10	
OAZ2	OSCAR	
OLMALINC	PHF19	
ORC4	BATF3	
OSBPL7	TPX2	
OSTF1	GBP4	
PAM	TRIM36	
PDHX	LILRB4	
PELO	SLFN12L	
PEX14	AC144652.1	
PGAM1	ASPM	
PHF3	PSRC1	
PLCXD2	SCCPDH	
PLGRKT	RHOC	
PMF1	CD38	
PMF1-BGLAP	SLA2	
POLR2F	HIST1H2AG	
POP4	PXDN	
PPA2	MTHFD1L	
PPCDC	CMC1	
PPFIBP1	GFI1	
PPID	ADAP1	
PPIL2	FOSL1	
PPP2R5A	TERC	
PPTC7	CENPO	
PRCC	SNORA71B	
PRKACB	STIL	
PRPF38B	ZAP70	
PRPF40A	CD33	
PSAP	GPRIN3	
PSMB9	TICRR	

QDPR	TYMS	
RAB12	NUCB2	
RAB35	P2RY6	
RANBP6	HJURP	
RB1CC1	DOK2	
RHOB	INSIG1	
RMND1	CDCA8	
RNASEH2B	ECI2	
RNASEH2B-AS1	CD96	
RNF138	CHEK1	
RNF2	PTGER4	
RNF6	USP13	
RNPEPL1	CD300E	
RPA3	CYP1B1	
RPL26L1	CDK6	
RPLP1	KB-1732A1.1	
RPS20	STOM	
RPS3	ECT2	
SACM1L	COL4A2	
SAMD12	LY6E	
SAT1	ABCB1	
SCAMP1	CEP41	
SCAMP1-AS1	CYSLTR2	
SCAMP2	MT-ATP6	
SCRN3	ARRB2	
SDCBP	RARRES3	
SDCBP2	E2F7	
SDCBP2-AS1	MIR3687	
SELENOS	GIMAP6	
SF3A3	FAM86JP	
SFT2D2	GPR171	
SGMS1	CTD-2510F5.4	
SGMS1-AS1	APOL1	
SLC16A7	SOAT1	
SLC30A1	PRKCQ	

SLC35B3	MEFV	
SLC41A2	HIST1H1D	
SMARCE1	RP13-735L24.1	
SMIM15	SNTB1	
SMIM15-AS1	LRFN4	
SNAP47	KIF21B	
SNORD54	C15orf39	
SNRPA	CDCA5	
SNRPA1	TDO2	
SP4	CMKLR1	
SPATS2L	C11orf84	
SPG21	CBX2	
SPIDR	TIMP3	
SPNS1	NBPF16	
SQOR	MSH5	
SRP14	AC004840.9	
ST6GALNAC3	SNORA71A	
STAM2	C18orf54	
STK38L	KIF2C	
SUB1	TROAP	
SUCLG2	PAICS	
SUCLG2-AS1	EMILIN2	
SUCO	PAQR6	
SUPV3L1	KIF21A	
SYF2	CD300C	
SYTL2	MOV10	
TAPBP	RNU11	
TBC1D1	HIVEP3	
TBC1D30	FAM86C1	
TBKBP1	CERS6	
TCTN1	RP11-686D22.10	
TES	AUNIP	
TET2	FMN1	
THEM4	SLC7A5P1	
THEMIS	RP11-932O9.10	

TIPARP-AS1	MDGA1	
TM9SF3	RP11-473M20.9	
TMBIM6	PPAT	
TMEM230	PFKP	
TMEM241	SMC4	
TMEM72-AS1	LRP8	
TMF1	XYLB	
TP63	UBE2C	
TRAF2	TOP2A	
TRAPPC8	ZYX	
TRIM25	CEP55	
TRIM33	UBE2T	
TRIM44	KIF23	
TRIP12	SPAG5	
TRPS1	POLQ	
TTC19	GBP1P1	
U2SURP	SAP30	
UBE3C	SLC29A1	
ULK3	DFNB31	
UMPS	FAM72B	
UPP1	FANCI	
UQCRB	BOP1	
UQCRFS1	FAM20C	
USP15	RP11-443B20.1	
UTP11	CDC25A	
WDR26	BUB1	
WSB1	LDLR	
XPNPEP1	PUS7	
XPO7	PRKCH	
ZBED5	FTL	
ZBED5-AS1	DBF4B	
ZBTB22	WARS	
ZCCHC6	LASP1	
ZCWPW2	PDE1B	
ZDHHC7	CMPK2	

ZFAT	LRRC8C	
ZFR	TRERF1	
ZNF281	SLC16A1	
ZNF558	FBXO41	
ZNF718	KIAA1524	
ZNF792	C16orf59	
ZNRD1ASP	YWHAH	
AADAC	NLGN4X	
AADACL2-AS1	HIST1H2BN	
ACOT9	OAS2	
ACOXL	FKBP5	
ACOXL-AS1	TSHZ3	
ACSL6	ARHGAP11A	
ACTL9	MS4A6A	
ADAM29	PXMP2	
ADAMTS1	C3AR1	
ADAMTS10	SBK1	
ADAMTS14	CENPE	
ADAMTS19	ALG3	
ADAMTS19-AS1	CDCA2	
ADAMTS5	TONSL	
ADD3-AS1	ECE2	
ADGRG1	DCLRE1B	
ADGRL3	DCLRE1A	
ADGRL3-AS1	LYAR	
ADORA2B	HIST2H2BE	
ADRA1A	KCNAB2	
ADRB1	MESDC1	
AGTRAP	GBAP1	
AHI1	STRIP2	
AIRN	NEIL3	
AKAP12	RAD54L	
ALDH8A1	CASC5	
ALKAL1	INPP4B	
AMZ1	FBXL19-AS1	

ANAPC1P1	PGAP1	
ANK2	PTPN7	
ANKRD36BP1	FADS1	
AOAH-IT1	FASN	
APBA2	MIR4435-1HG	
ARHGAP26-AS1	GBP2	
ARL6IP4	FKBP4	
ARNTL2	ENC1	
ASAH2	ERCC6L	
ASAP3	HSP90AB1	
ASZ1	PA2G4	
ATP8A2	ITPK1	
ATP9A	RRM2	
B3GALT1	UBE2L6	
BBS12	NCAPH	
BCR	RNU6ATAC	
BICDL1	GCH1	
BMP2	FAR2	
BVES-AS1	RP11-427H3.3	
C10orf55	IL15	
C16orf97	TFR2	
C1orf100	PSTPIP1	
C1orf140	C2orf48	
C1orf226	TSHZ1	
C20orf203	FEN1	
C2orf92	CYSTM1	
C3	LPAL2	
C3orf35	RNF213	
C4orf45	FAM102B	
C4orf51	SLC16A6	
C9orf106	SPC24	
C9orf92	RASGEF1A	
CA10	DPY19L2P2	
CA5A	SNORD104	
CACNA1E	SUV39H2	

CACNG4	PXN	
CACNG5	GDF11	
CAMK2G	FPR1	
CASC20	DPF3	
CASC22	APOL3	
CASS4	STX11	
CBLN2	FAM72D	
CCDC68	PROB1	
CCDC80	XRCC6BP1	
CCL1	VIM	
CCL11	CIT	
CCL15	WDHD1	
CCL15-CCL14	NME1	
CCL7	MPP1	
CCL8	TUBB	
CD200R1L	PLAU	
CD300A	SLC25A53	
CD34	HIRIP3	
CD36	MTHFD1	
CD70	LARP1	
CDC20B	SRI	
CDCP1	ZNF280B	
CECR3	NCR3LG1	
CETN4P	WDR54	
CFAP45	CMTM3	
CFTR	PLA2G7	
CHST2	STAT1	
CHSY3	GNPNAT1	
CLEC2A	C5orf30	
CLEC3B	EME1	
CLIC5	PNPO	
CLNK	IFIT3	
CLRN2	PIGW	
CNBD1	KTN1-AS1	
CNTN4	RPS6KA3	

CNTN4-AS2	ARMC5	
CNTN6	SLC20A1	
COL14A1	SPATA13	
COL4A2-AS1	GSG2	
COLQ	LMNB1	
CORT	VCAN	
COX6A2	SHROOM1	
CPLX3	TSEN54	
CRAT37	CTSC	
CRYBA4	GTSE1	
CRYBB1	CELF2	
CRYBB3	MAPKAPK3	
CSF2	CHAMP1	
CSMD3	SH3BP1	
CTD-2350J17.1	SPRY4	
CTNNA2	PLK4	
CTXND2	TUBA1B	
CX3CR1	HELLS	
CXADRP2	ATAD2	
CYYR1	RP11-713M15.1	
DACH1	BCAT1	
DCAF4L2	E2F8	
DDIT4L	SYTL3	
DEPP1	GSK3A	
DHRS9	FSD1	
DINOL	POLR1A	
DLEU7-AS1	FXN	
DMBT1	FAM216A	
DMD	ADAP2	
DNER	KNSTRN	
DRAIC	DLGAP5	
DYNAP	FOXRED2	
EDAR	RACGAP1	
EDF1	KIF11	
EDN1	MYADM	

EEF1DP3	PM20D2	
EIF1AX-AS1	RP11-61A14.3	
EIF2AK4	PFAS	
ELFN2	NFAM1	
ENO3	GIMAP5	
EPHA1	MELK	
EPHB3	RP11-1094M14.9	
ERCC1	ANTXR2	
ESM1	NFATC3	
ESRRG	APOC1	
EVADR	ZCCHC3	
EVI5	NCAPG	
EYA4	COMMD7	
EYS	LINC00422	
FAM184A	WDR62	
FAM198A	TMEM201	
FAM19A1	TRIP13	
FAM20A	RNF130	
FAM47A	GPR19	
FAM84A	HAS2	
FAT3	DUSP4	
FBXL7	SLAMF7	
FBXW4P1	PNMA1	
FER	DSCC1	
FERMT1	CSNK2B	
FEZ2	ABCE1	
FFAR4	NCAPG2	
FGD4	RHEBL1	
FGF5	ALDH1B1	
FKBP8	DAB2	
FMNL2	ATP8B3	
FNIP2	JAKMIP2	
FOXG1-AS1	GALM	
FOXP2	PKMYT1	
FREM2	UBE2S	

FREM2-AS1	ATP1A3	
FRY	MICB	
FRY-AS1	CCDC86	
FXYD2	CDC25B	
FXYD6	SNORA31	
FXYD6-FXYD2	GLUL	
GACAT2	ZADH2	
GADD45G	CCNA2	
GALNT17	EXO1	
GALNT8	PTPN22	
GCNA	RP11-274E7.2	
GGNBP1	MYO7A	
GHRLOS	C20orf112	
GLI3	CENPV	
GNAI1	RUNX1	
GNAT3	DMXL2	
GPR27	SLC19A1	
GPR87	PYCR1	
GPRC5C	CENPI	
GPX8	CHTF18	
GRIK4	NCL	
GRM5	GMPS	
GRM5-AS1	CBLB	
GTSF1L	AC116366.5	
GUCA1A	KDELC1	
GUCA2B	RRP9	
GUCY1B2	AGO2	
HABP4	KIF4A	
HAVCR1	CTPS1	
HCG20	FADS2	
HDC	ACAA2	
HEATR9	SLX4IP	
HECW1	MLKL	
HFM1	KSR1	
HGF	RP11-644F5.11	

HKDC1	DEPDC1B	
HLX	LIN7A	
HLX-AS1	ME2	
HMOX1	FAM127B	
HS3ST4	CD14	
HS6ST3	FAM72A	
HSD17B3	MCM8	
HSD52	TTYH3	
HSPA4L	ERI1	
HSPA7	SGOL1	
HTRA1	SLC43A3	
HTT	PATL1	
HULC	CCSAP	
IER3	UBASH3A	
IER3-AS1	NDC80	
IFITM10	NCAPD3	
IFNG-AS1	ATN1	
IL18R1	PPRC1	
IL18RAP	HOXB3	
IL21	LRRC25	
IL21-AS1	SNORA79	
IL22	GPATCH4	
IL26	KPNA2	
IL3	GPR84	
ILDR1	NPL	
INHBA	WRNIP1	
INHBA-AS1	HSPD1	
INSRR	NACC1	
INTU	AIF1	
IQCN	OAF	
ISYNA1	RAD51D	
ITGA2	TDRD6	
ITGB5	B4GALT5	
ITLN1	RNU2-61P	
ITM2A	STARD3NL	

ITPK1-AS1	CHAF1B	
JAG1	C9orf89	
JPH4	RP11-452L6.5	
KCNA1	XCR1	
KCNA6	WAS	
KCNJ12	NUF2	
KCNJ15	C20orf27	
KCNJ18	MIR181A1HG	
KIAA1024L	ABCA1	
KIAA1671	MAFB	
KIAA2026	CENPA	
KIF13B	TGM2	
KIF3C	NOP2	
KLRA1P	PLK1	
KRT222	CKAP5	
KRT71	APOL6	
KRT72	ITK	
KRT74	40787	
KTN1	SLFN11	
LAMA2	COA7	
LDB2	COL15A1	
LDLRAD3	ANP32E	
LHFPL2	ZNF367	
LIM2	SNHG17	
LINC00092	BRCA2	
LINC00189	C19orf54	
LINC00222	ALG8	
LINC00243	EAF1	
LINC00290	ITGA5	
LINC00298	TMEM164	
LINC00299	SUV39H1	
LINC00336	APOL2	
LINC00348	PFN1	
LINC00355	RP11-402J7.2	
LINC00359	LAMB1	

LINC00376	C9orf40	
LINC00395	C20orf24	
LINC00415	TRPV2	
LINC00433	CDC7	
LINC00437	CHAF1A	
LINC00440	KHDC1	
LINC00462	ZNF473	
LINC00491	PSME2	
LINC00506	SLCO4A1	
LINC00520	CXCR3	
LINC00536	RP11-10O17.1	
LINC00560	ZMIZ1	
LINC00563	XPO5	
LINC00589	IGSF6	
LINC00593	RNU2-59P	
LINC00645	RP11-485G4.2	
LINC00689	ZNRF1	
LINC00706	CKAP2L	
LINC00905	CLPB	
LINC00919	TTK	
LINC00943	HNRNPLL	
LINC00944	GINS4	
LINC01010	HOXC4	
LINC01023	ZWINT	
LINC01047	RP4-800G7.2	
LINC01080	DDX11	
LINC01140	RRS1	
LINC01146	SLC46A1	
LINC01227	NRP1	
LINC01260	ZKSCAN2	
LINC01267	CENPJ	
LINC01279	CEP152	
LINC01331	GAB3	
LINC01333	GAS7	
LINC01335	CCT5	

LINC01344	GLIPR2	
LINC01349	ATAD5	
LINC01352	KIF20B	
LINC01364	CCDC65	
LINC01370	TUBG1	
LINC01428	CASC7	
LINC01449	UCK2	
LINC01482	AC093616.4	
LINC01503	TOMM40L	
LINC01508	SLA	
LINC01532	CHSY1	
LINC01571	ATF5	
LINC01625	RNASEH1-AS1	
LINC01633	ZMYM3	
LINC01693	ISG20L2	
LINC01694	BST2	
LINC01699	LRIF1	
LINC01713	TNIK	
LINC01728	SPDL1	
LINC01751	SAMHD1	
LINC01759	C17orf53	
LINC01762	MDC1	
LINC01776	E2F1	
LINC01790	MSN	
LINC01804	PRR3	
LINC01814	TRIM14	
LINC01815	DGKZ	
LINC01821	FXYD5	
LINC01840	CASP2	
LINC01844	YARS	
LINC01852	NUDT19	
LINC01899	TNFRSF12A	
LINC01950	FAM207A	
LINC01973	NLN	
LINC01980	NOP56	

LINC01981	ENO1	
LINC01987	RGL4	
LINC02015	HNRNPAB	
LINC02042	CTD-3099C6.9	
LINC02054	RNU12	
LINC02057	AVEN	
LINC02069	SLC9B2	
LINC02072	POLR1B	
LINC02073	C2orf44	
LINC02099	LMNB2	
LINC02127	FOLR2	
LINC02149	PRC1	
LINC02180	FBXO5	
LINC02345	CACNB1	
LINC02367	RBMS1	
LINC02446	DNA2	
LINC02493	CHST11	
LINC02495	SMC2	
LINC02497	DONSON	
LINC02500	URB2	
LINC02556	RP5-1024G6.8	
LINCMD1	STIP1	
LINP1	GAS5-AS1	
LIPM	RFWD3	
LMNB1-DT	AGPAT1	
LMX1B	CDC25C	
LOC100128317	COX10	
LOC100128531	KIF18A	
LOC100128988	EIF2AK2	
LOC100129291	PSME3	
LOC100129297	INCENP	
LOC100129316	CD99	
LOC100131604	KIF24	
LOC100192426	CHST12	
LOC100287036	TMX4	

LOC100288911	NT5DC3	
LOC100506207	TBC1D31	
LOC100506271	ARHGAP11B	
LOC100506274	FPR3	
LOC100507468	CMSS1	
LOC100996671	HSPH1	
LOC101927269	AAK1	
LOC101927378	APMAP	
LOC101927543	NCALD	
LOC101927822	MT-ND4L	
LOC101927844	MYB	
LOC101927847	GPR65	
LOC101927914	RAB27A	
LOC101928021	RP11-10K16.1	
LOC101928030	HPRT1	
LOC101928053	CDK2	
LOC101928231	ATXN2L	
LOC101928791	EMP3	
LOC101928988	SCN1B	
LOC101928994	DVL3	
LOC101929116	SLC5A3	
LOC101929188	SUPT16H	
LOC101929227	CFH	
LOC101929331	G6PD	
LOC101929353	POLD1	
LOC101929452	SLC36A1	
LOC101929549	FYB	
LOC101929551	TMEM185B	
LOC101929709	GIMAP2	
LOC102467080	CUL1	
LOC102467081	FAM86A	
LOC102723968	LGMN	
LOC102724434	E2F2	
LOC105369431	TRAIP	
LOC105369532	PDCD11	

LOC105370306	PTPRJ	
LOC105372397	RNU5B-1	
LOC105376975	ACOT2	
LOC105377975	CERS2	
LOC105616981	BAZ1B	
LOC150935	DKC1	
LOC153910	MAP7D3	
LOC158434	WHSC1	
LOC158435	LIG3	
LOC284395	GTF3C6	
LOC285762	MEX3D	
LOC285766	NOM1	
LOC339862	ZBTB2	
LOC339902	APOE	
LOC400800	BRPF3	
LOC440084	KIAA1958	
LOC646214	RGS3	
LOC728715	RECQL4	
LOC728755	MAPRE1	
LOC729603	ANPEP	
LOC729815	NAT10	
LOC90768	FAM105A	
LRIG1	IFITM3	
LRRC20	SNORA76	
LRRC37A8P	MYRF	
LRRN3	SLC7A5	
LRRTM4	KLC2	
MAGI2	SF3B4	
MAP7D2	ASUN	
MARC1	E2F3	
MAS1	TAF5	
ME3	RNU5A-1	
MED4-AS1	BAX	
MFN2	CENPN	
MGC34796	RASSF1	

MIR1183	MAGOHB	
MIR1234	RP11-403I13.8	
MIR133B	CCDC85B	
MIR137HG	RAD23B	
MIR195	MNDA	
MIR199A1	C21orf58	
MIR2053	SGOL2	
MIR206	STAB1	
MIR3118-2	DRAM1	
MIR3118-3	CHCHD4	
MIR3118-4	GK5	
MIR3136	DTX3L	
MIR3138	NR1H3	
MIR3171	SACS	
MIR326	BIVM	
MIR340	CACYBP	
MIR34AHG	C17orf96	
MIR3614	CTD-2547L24.3	
MIR3657	GNL3L	
MIR3662	CCDC150	
MIR3914-1	PLAGL2	
MIR3973	RASSF4	
MIR422A	CENPL	
MIR4259	SRPK1	
MIR4265	DIAPH3	
MIR4266	LINC00883	
MIR4270	GNS	
MIR4290	ARHGDIA	
MIR4290HG	AC079922.3	
MIR4304	DCAF7	
MIR4460	MAZ	
MIR4473	MTR	
MIR4474	H1FX	
MIR4479	BLM	
MIR4493	LRRCC1	

MIR4501	EIF4A1	
MIR4522	SNHG3	
MIR4529	SLC8A1	
MIR4534	MRM1	
MIR4665	ARSG	
MIR4759	RRP12	
MIR4771-1	CCDC77	
MIR4771-2	ATXN1	
MIR4772	ZNF438	
MIR4776-1	NUP107	
MIR4776-2	GIT1	
MIR4777	RAD23A	
MIR4789	FGL2	
MIR497	MT-CO2	
MIR5192	BRCA1	
MIR544B	SLC35B4	
MIR548H2	SLFN5	
MIR548J	PTGES3P1	
MIR548W	C10orf2	
MIR556	ESPL1	
MIR5680	ZNF609	
MIR579	F8A1	
MIR6088	C14orf80	
MIR612	RGL1	
MIR6124	CALU	
MIR634	HK2	
MIR6729	PSMC3IP	
MIR6882	PIF1	
MIR6892	GSE1	
MIR7843	TRGV4	
MIR887	HCFC1	
MLANA	ARID5A	
MMP16	CTC-503J8.6	
MOAP1	PRKACA	
MPZL2	TOP3A	

MS4A4E	NCOA1	
MS4A6E	CSF1	
MSANTD1	NUDT6	
MTNR1B	LBX2-AS1	
MTVR2	PPARGC1B	
MYBL2	KHSRP	
MYH13	APOO	
MYH15	SPRED1	
NAALADL2	B3GNTL1	
NAALADL2-AS1	RAB20	
NAALADL2-AS2	CAD	
NAALADL2-AS3	ITGAL	
NAXD	LPCAT4	
NBAS	CD2	
NBPF4	METRNL	
NBPF6	PDCD1LG2	
NCAM2	ARRB1	
NCOR2	POLE	
NCRUPAR	AGPS	
NECTIN4	AATF	
NELL2	MSANTD3	
NETO1	SFXN3	
NEURL3	TTLL3	
NID2	IL15RA	
NINJ1	HLA-H	
NKAIN2	ZNF280C	
NOS1AP	PTPLAD1	
NPBWR1	AC009948.5	
NPDC1	NCKIPSD	
NPEPPS	ARAP2	
NPM2	ARID1A	
NPR3	SIMC1	
NRON	RAP2B	
NT5DC4	RCN1	
NTM	PREP	

NUTM1	TMEM194A	
NYAP2	CCND2	
OPTC	MTHFD2	
OR2B2	PTMS	
OR2Z1	RIN3	
OR7E156P	TMCO6	
OXGR1	DENND1B	
P2RY14	ESYT2	
P3H2	SLC38A7	
PALD1	ATP2A2	
PANDAR	ADAT2	
PAX8	TLN1	
PAX8-AS1	CENPK	
PCAT29	SMYD2	
PCBP3	PRIM2	
PCDH7	NAIP	
PCDH9	SP2	
PCDH9-AS4	RPL26	
PCGEM1	SAMD9L	
PDE6H	TCF19	
PEX5L	ADSS	
PHTF2	CTA-384D8.36	
PKD2L1	KNOP1	
PLCXD2-AS1	MYCBPAP	
PNMA2	IRF1	
POPDC3	YPEL1	
POU3F1	EFCAB4B	
PPARG	SLC2A13	
PPP1R2B	PWP2	
PPT1	RAB3IL1	
PRKAR1A	VPRBP	
PRKCA	RUSC1-AS1	
PRKCA-AS1	AEN	
PROKR1	COLGALT1	
PROX1	C4orf48	

PROX1-AS1	FADD	
PRRC2B	ANKRD32	
PRTFDC1	PELP1	
PRUNE2	TOPBP1	
PTH1R	PGPEP1	
PTHLH	EXOSC6	
PTPN14	HELZ	
PTPRK	IDH2	
PTPRM	PGP	
PTPRN2	UBXN2B	
PXN-AS1	SRRM1	
RAB27B	ZFYVE28	
RAPGEF4	ANLN	
RASD2	MTBP	
RASGEF1C	SUPT3H	
RBM24	PLP2	
RBPMS2	EMILIN1	
RCAN1	LIMK1	
REP15	C19orf55	
REREP3	PRRC2A	
RET	RTP4	
RGS12	DDB2	
RGS6	EHBP1L1	
RIMS1	RFX7	
RIPK2	DNAJA1	
RMRP	CABIN1	
RNF144A	SRM	
RORA-AS2	ICMT	
RPL13AP17	FSTL3	
RPL13AP3	SSH1	
RXFP2	NCF2	
RYR1	PAXIP1	
S100B	RAC2	
SALL1	E2F6	
SAP25	MMS22L	

SATB2-AS1	ZKSCAN5	
SCARNA21	TTI1	
SCARNA9L	FLNA	
SCG3	YEATS2	
SCOC	AGAP3	
SCOC-AS1	AP3M2	
SEMA3A	AP2B1	
SEMA3E	ITGA4	
SEPT10	G6PC3	
SERPINE2	AKT1	
SFTPB	POGK	
SGSM1	POLA1	
SH3RF3	PSMB10	
SH3RF3-AS1	NFATC2	
SHISA2	PBDC1	
SIGLEC7	TPP1	
SIT1	ETFB	
SLC14A1	SLC16A1-AS1	
SLC14A2	MBD6	
SLC15A2	DHX33	
SLC25A31	C9orf41	
SLC26A11	ALDH3B1	
SLC35D2	LAP3	
SLC36A4	RRP1B	
SLC9A4	WNK1	
SLC9A9	SIGMAR1	
SLC9A9-AS1	DFFA	
SMAD1	TRIM56	
SNORA38B	IFI16	
SNORA75B	MCTP2	
SNORD105	MAPK1IP1L	
SNORD129	DNAJC9	
SNORD151	ZNF142	
SNORD154	TGFB1	
SNORD62A	MDM2	

SNORD62B	FLVCR1	
SNORD82	CTDNEP1	
SNX7	APOBEC3C	
SORCS1	TRAPPC1	
SOX10	KPNA3	
SPATA46	PLEK	
SPOCK3	RP1-151F17.2	
ST18	RAD54L2	
STOML3	ABI2	
SV2B	ANKRD28	
SYCP3	SKAP1	
SYN2	LINC00152	
SYT6	BMP1	
SYT8	RNF26	
TBC1D9B	TOR2A	
TBX19	HNRNPA2B1	
TCF4-AS1	ZNF777	
TECRL	CEP85	
TEDDM1	TMCC1-AS1	
TEMN3-AS1	B3GALT6	
TENM1	CEP78	
TESC-AS1	C4orf21	
TET2-AS1	POMGNT2	
TEX13D	SLC29A3	
TEX48	POLR3D	
TEX53	ODF2	
THBS1	CCDC41	
TIMD4	MLLT1	
TIMP4	ATP2B4	
TLL2	MT-CO3	
TMCO5A	WIZ	
TMEFF2	HNRNPUL1	
TMEM158	FBF1	
TMEM176A	EHMT2	
TMEM200A	SNX27	

TMEM211	ARHGAP30	
TMEM244	LRRC61	
TMEM26-AS1	ADAR	
TMEM52B	TCERG1	
TMEM72	ZEB1-AS1	
TMIGD1	NUP62	
TNFSF18	TOMM5	
TNFSF9	NEU3	
TNRC6C	DCTD	
TPRG1	PPP2R5E	
TPRG1-AS1	SRGAP2	
TPRG1-AS2	CEP57L1	
TUBB4A	BTN3A2	
TUSC8	ARL4C	
TXNDC16	RP11-441O15.3	
UBE2E2-AS1	LIPG	
UGGT2	SNORA26	
UNQ6494	CTSZ	
VCAM1	DAB2IP	
VENTXP7	PAM16	
VGLL3	MT-ND1	
VIPR2	ZBED6	
VIT	FANCG	
VSTM2B	LTV1	
WASHC2A	FRMD5	
WDFY1	MLEC	
WDR1	CNTLN	
WNT2	TCOF1	
WSCD2	CBL	
XIRP1	UBALD2	
ZBTB17	CBX6	
ZFAT-AS1	TAP1	
ZFP69B	SPATA5	
ZFR2	TADA3	
ZMYM4	TMEM143	

ZNF385D	CST3	
ZNF385D-AS1	HNRNPA3	
ZNF385D-AS2	CPVL	
ZNF407	GBP3	
ZNF414	ESF1	
ZNF518B	PARVB	
ZNF595	PIK3R3	
ZNF843	AP5B1	
ZSWIM6	HSP90AA1	
ZSWIM7	HLA-A	
	RP11-22B23.1	
	KIAA1467	
	FBXL6	
	TBC1D2B	
	PROSER1	
	TMPO-AS1	
	RBPJ	
	APOBEC3D	
	STARD4	
	IFI35	
	RIF1	
	KNTC1	
	SMC1A	
	TAP2	
	CENPP	
	LGALS9	
	PRR5L	
	SNHG12	
	DENND2D	
	FAM111A	
	GAR1	
	RPRD2	
	CTSD	
	TMEM135	
	AGTPBP1	

	EPB41L3	
	ARID3B	
	RNPS1	
	FYN	
	NCOA6	
	CNTROB	
	AP2A2	
	GALNT10	
	MEF2D	
	DPYSL2	
	S100PBP	
	TAF9B	
	PDE4D	
	DHX30	
	RTF1	
	PHTF1	
	SLC35A4	
	NMI	
	RPL36A	
	ST3GAL5	
	HNRNPU	
	C22orf46	
	CTAGE5	
	DARS2	
	SNORA37	
	AHSA1	
	IL2RG	
	CLEC10A	
	POGZ	
	BRICD5	
	MIR3648	
	RAB11FIP3	
	FAM178A	
	DCUN1D3	
	ST3GAL1	

	BTN2A3P	
	KIAA0368	
	SLC4A2	
	ARSB	
	OTUD7B	
	SLC1A5	
	TIPARP	
	RP11-448A19.1	
	DOT1L	
	C2CD2L	
	EXOSC9	
	PML	
	TPST2	
	STX2	
	SELPLG	
	DPYD	
	SOCS7	
	UBAP2L	
	PTPN9	
	TTL	
	ATXN2	
	ARF3	
	ADAT1	
	KLHDC4	
	AXIN1	
	UHRF1BP1	
	C5orf58	
	FIGNL1	
	SNTB2	
	PUS1	
	PSMA6	
	REXO1	
	IPO9	
	DENND1A	
	MACF1	

	SUV420H2	
	BRI3	
	WIPF1	
	EXOC2	
	MCOLN1	
	PLB1	
	MED1	
	ARHGAP33	
	FANCB	
	ENDOD1	
	UBFD1	
	MPHOSPH9	
	TET3	
	APOBEC3F	
	NASP	
	NTNG2	
	TAGLN2	
	ZCCHC2	
	IDS	
	CECR1	
	AGPAT3	
	PCNT	
	BRE	
	ACP2	
	SYNJ1	
	WDR90	
	HIPK1	
	HSF4	
	MB21D1	
	KLF16	
	TRIM21	
	ADCY7	
	ZNF384	
	LARS2	
	PLEKHO2	

	FNBP1P1	
	CAMTA2	
	TAF4	
	SLC25A44	
	PLEKHB2	
	HLA-B	
	PTP4A1	
	SEMA4D	
	HEATR3	
	ABL2	
	AC073046.25	
	ANKRD52	
	ATIC	
	PRRC2C	
	TBC1D10B	
	TFEC	
	AOAH	
	ZNF746	
	DECR2	
	SIK3	
	ASCC3	
	CHKA	
	SRCAP	
	SRGAP2B	
	RASL12	
	TOR1A	
	SNX18	
	PARP9	
	SETD1A	
	FAM89B	
	HYOU1	
	SLC19A2	
	CALR	
	TMSB15B	
	FAM120A	

	FAM211A	
	TEX261	
	FAM50A	
	CD247	
	NUP214	
	VPS26B	
	C17orf89	
	KIAA0895L	
	ACSL4	
	AP1S2	
	TRIM11	
	ITSN1	
	TTLL4	
	MGRN1	
	RHBDD3	
	GPRIN1	
	CYBB	
	SAP130	
	BRD8	
	FAM193A	
	HUWE1	
	RP11-571M6.8	
	ANP32B	
	FKBP15	
	R3HDM1	
	SLC25A37	
	NUPL1	
	PTPN12	
	NCOA5	
	PARP4	
	GALC	
	IL10RA	
	ANAPC1	
	GDI1	
	EIF4G3	

	ZC3H4	
	NUP188	
	OPA3	
	ETV5	
	CD99L2	
	SPATA20	
	DPY19L1	
	INPPL1	
	CYTH4	
	EIF4EBP2	
	ELF4	
	ZBED4	

Supplemental Table S5. shRNA screening identifies shared downregulated, upregulated genes affected by TOX2-shRNA1 and TOX2-shRNA2 in NKYS cell.

**shTOX2 vs shScr (Downregulation)**	**shTOX2 vs shScr (Upregulation)**
LIMCH1	IGF1
ZP2	ARHGAP31
DPYSL3	IFT27
TOX2	KLHL4
FCRLA	HAGHL
LGR6	CHPT1
FBLN2	MDN1
KRT80	KYNU
SCARA5	ELOVL4
CDH4	CMPK2
PRR5	CYP4V2
MICA	LHFPL2
HLA-DPB1	PSPH
ARL14EPL	CD226
AC130304.1	SLC7A11
TFAP2B	PDE3B
ATP12A	ANKRD22
LGALS3BP	CCR5
MDGA1	CCDC78
KLHL24	TRAT1
SLAMF1	TIGD6
KRT7	GAS1
CCNG2	MB21D2
DMP1	PMCH
IP6K3	TPRG1
PRKCE	SELL
ACBD7	TCF4
ALOX12B	CARD11
GJD3	F5
H2AC18	VIT
CFI	ADGRG1
CCDC13	SLC12A8
CALCOCO1	SOX2-OT
DPEP1	AC005831.1
ERO1B	AC244258.1
NDRG1	ABBA01000935.2
LILRB1	NEFH
STAG3L4	APOBEC3H
RASSF4	SASH1
CILK1	COX7A2

SPP1	IL10
YPEL5	LINC00467
CD244	FBN1
SERPINB6	ZNF239
PIMREG	SVIL
KLC4	SNX18P3
SULF1	TNC
GABARAPL1	TIE1
ITGB7	RASSF2
CGN	RIC8B
TBC1D7	IL18RAP
STC1	AOAH
ISG20	ELAC1
STARD5	MYO1F
YPEL2	RGS18
PTP4A3	AUTS2
IFITM1	CYB5A
SNHG32	KATNAL2
HLA-C	MYO5B
HLA-F	TLR6
HCP5	GPC6
HLA-A	EVI2B
HCG11	ZSCAN30
HLA-B	LINC00638
FRG2EP	RMRP
	RPPH1

Supplemental Table S6. TOX2 binding on 12 genes out of 65 genes downregulated by TOX-shRNA.

Gene	Number of ChIP-seq binding site	Binding region
LIMCH1	5	intron (NM_001289122, intron 2 of 25)
		**promoter-TSS (NM_001289124)**
		intron (NM_001289122, intron 1 of 25)
		Intergenic
		intron (NM_001289122, intron 1 of 25)
ZP2	1	intron (NM_003460, intron 10 of 19)
DPYSL3	4	intron (NM_001197294, intron 1 of 13)
		3'-UTR (NM_001287740, exon 14 of 14)
		intron (NM_001197294, intron 1 of 13)
		Intergenic
TOX2	2	intron (NM_001098797, intron 3 of 8)
		intron (NM_001098797, intron 3 of 8)
FBLN2	1	intron (NM_001004019, intron 2 of 17)
KRT80	2	intron (NM_182507, intron 2 of 8)
		Intergenic
TFAP2B	2	Intergenic
		Intergenic
PRKCE	2	intron (NM_005400, intron 1 of 14)
		intron (NM_005400, intron 1 of 14)
GJD3	1	intron (NM_001024809, intron 6 of 7)
NDRG1	1	intron (NM_001258432, intron 1 of 13)
YPEL2	1	Intergenic
PTP4A3	1	**promoter-TSS (NM_032611)**

Supplemental Table S7. Identification of TOX2 binding motif (^VSSSGVVGCG) in PTP4A3 promoter.

Motif_alt_id	Sequence_name	Start*	Stop*	Ccore	p-value	q-value	Matched_sequence
VSSSGVVGCG	PTP4A3_chr8:141390021-141394021	-113	-104	12.9515	2.17E-05	0.0252	GGGGGCGGCG
VSSSGVVGCG	PTP4A3_chr8:141390021-141394021	-107	-98	12.9515	2.17E-05	0.0252	GGCGGGCGCG
VSSSGVVGCG	PTP4A3_chr8:141390021-141394021	-99	-90	12.9515	2.17E-05	0.0252	CGCGGCCGCG
VSSSGVVGCG	PTP4A3_chr8:141390021-141394021	-96	-87	12.9515	2.17E-05	0.0252	GGCCGCGGCG
VSSSGVVGCG	PTP4A3_chr8:141390021-141394021	-13	-14	12.9515	2.17E-05	0.0252	CGCGGCCGCG
VSSSGVVGCG	PTP4A3_chr8:141390021-141394021	+90	+99	12.9515	2.17E-05	0.0252	GGCGGGGGCG
VSSSGVVGCG	PTP4A3_chr8:141390021-141394021	+92	+101	12.9515	2.17E-05	0.0252	CGGGGGCGCG
VSSSGVVGCG	PTP4A3_chr8:141390021-141394021	+97	+106	12.9515	2.17E-05	0.0252	GCGCGCGGCG

^ IUPAC notation: V for C/G/T; S for C/G

Start* to Stop*: position relative to transcription start site (TSS)

Supplemental Table S8. RUNX3 binding motifs on TOX2-SE and their ChIP-PCR primers.

RUNX3 binding site	Motif position	Primer sequences
E1	chr20:42568538-42568545	F1: 5'- TGCCTGGCACAGAGTAGATG -3'<br>R1: 5'- GCATGACTCCCTGCCCTTTA -3'
E2	chr20:42579003-42579010	F2: 5'- GAGGTTCCTCGGATGTGCTG -3'<br>R2: 5'- CCCCAGAAAGAGGAAGCCAG -3'
E3	chr20:42588335-42588342	F3: 5'- TCATTGTCTCAGTCACACTCGT -3'<br>R3: 5'- TAACTAAAAGCCGCATGAGCG -3'

Supplemental Table S9. Clinical features of 42 cases of NKTL and their expression of TOX2, RUNX3 and PRL-3 in CD3+ NKTL tumor cells.

UPN	Age (y)	sex M=1 F=0	Ethnicity	Diagnosis	% of CD3+ cells	Average of OPN Mean Intensity	Average of PRL-3 Mean Intensity	Average of RUNX3 Mean Intensity	Average of TOX2 Mean Intensity	Over survival (months)	therapeutic Regime
UPN1	65	1	Chinese	extranodal NKTL	90.61%	7.394284	3.002738	37.26697	1.466337	4.13	CHOP + RT
UPN2	33	NA	NA	extranodal NKTL	80.98%	7.327955	2.5666	28.23033	0.930459	NA	NA
UPN3	45	1	Chinese	extranodal NKTL	68.17%	6.194917	1.067165	23.89497	0.222303	263.4	CHOP + RT
UPN4	69	1	Chinese	extranodal NKTL	46.92%	5.093111	0.874702	28.39717	0.147577	NA	NA
UPN5	46	1	Chinese	extranodal NKTL	63.82%	3.369431	0.879413	21.40294	0.308685	4.57	CHOP
UPN6	51	1	Chinese	extranodal NKTL	72.64%	8.596648	1.202651	30.31302	0.177647	6.6	SMILE
UPN7	82	1	Chinese	extranodal NKTL	59.97%	5.002496	1.360308	14.65681	0.002767	NA	NA
UPN8	17	1	Chinese	extranodal NKTL	71.90%	5.496517	0.945192	32.5889	0.157369	3.53	FLU
UPN9	47	1	Chinese	extranodal NKTL	35.15%	3.899534	0.60208	23.86307	0.148119	7.9	CHOP --> DHAP
UPN10	32	1	Malay	extranodal NKTL	61.52%	4.715732	1.149132	19.63255	0.203751	NA	NA
UPN11	46	0	Chinese	extranodal NKTL	52.46%	4.511232	0.599461	11.3639	0.03694	NA	NA
UPN12	52	1	Chinese	extranodal NKTL	61.47%	5.565914	1.188073	20.03456	0.178141	NA	NA
UPN13	62	1	Chinese	extranodal NKTL	59.48%	5.389707	1.110743	21.70389	0.202325	3.57	RT
UPN14	56	1	Chinese	extranodal NKTL	60.01%	4.775464	1.369269	24.40323	0.229999	NA	NA
UPN15	29	0	Indonsian	extranodal NKTL	32.46%	6.963221	0.548787	22.77629	0.078642	44.8	RT
UPN16	26	1	Chinese	extranodal NKTL	59.09%	6.226929	0.975057	28.95109	0.630805	0.53	NA
UPN17	32	1	Chinese	extranodal NKTL	58.77%	9.396764	1.222021	25.91712	0.192903	62.7	ICE + RT
UPN18	37	1	Chinese	extranodal NKTL	43.17%	6.157622	0.619345	18.43302	0.082614	37.07	GIFOX-B + RT
UPN19	52	1	Pakistani	extranodal NKTL	72.45%	7.191923	1.01087	22.97384	0.125467	4.63	RT
UPN20	58	0	Chinese	extranodal NKTL	58.43%	4.642634	1.598177	22.24805	0.333798	25.87	SMILE + BMT
UPN21	55	1	Chinese	extranodal NKTL	35.15%	4.561414	0.949125	26.82183	0.308786	11.2	ICE + RT
UPN22	65	1	Chinese	extranodal NKTL	48.78%	5.569317	1.838015	26.80178	0.105642	2.23	m-BACOD
UPN23	60	0	Chinese	extranodal NKTL	55.72%	9.541487	1.153713	27.31641	0.251937	5.87	GIFOX-B
UPN24	65	1	Malay	extranodal NKTL	68.81%	6.659114	1.106123	25.54481	0.740558	0.4	NA
UPN25	40	1	chinese	extranodal NKTL	59.28%	4.563301	1.513984	20.42494	0.387156	0.4	NA
UPN26	39	0	Chinese	extranodal NKTL	48.95%	5.474348	1.01763	21.58534	0.195355	82.87	SMILE + RT
UPN27	68	0	Chinese	extranodal NKTL	60.16%	5.742883	0.628603	18.14864	0.12567	1.33	L-ASP, ETP
UPN28	65	0	Chinese	extranodal NKTL	57.38%	6.469747	1.290395	25.64853	0.229245	78.77	SMILE + RT
UPN29	51	0	NA	extranodal NKTL	69.87%	9.456637	0.79612	27.03723	0.16775	NA	NA
UPN30	54	1	Chinese	extranodal NKTL	72.50%	4.896238	0.850453	23.95717	0.24257	146.47	SMILE + BMT
UPN31	69	0	Chinese	extranodal NKTL	58.19%	4.030339	0.931664	26.1669	0.879483	0.007	VCR + PSL + HCT
UPN32	70	0	Chinese	extranodal NKTL	56.08%	4.963332	1.326705	24.61369	0.178749	1.83	CT (others)
UPN33	61	1	Chinese	extranodal NKTL	55.02%	3.693718	0.795721	22.2676	0.183336	61.3	SMILE + RT
UPN34	53	1	NA	extranodal NKTL	74.38%	5.565899	1.098456	27.9402	0.060667	NA	NA
UPN35	61	1	Chinese	extranodal NKTL	57.84%	6.048051	1.37289	28.06425	0.35535	55.4	ICE + RT
UPN36	30	1	Chinese	extranodal NKTL	62.83%	5.550599	1.006421	29.12791	0.417022	14.63	SMILE
UPN37	54	1	Chinese	extranodal NKTL	62.90%	6.121998	0.803613	24.22935	0.233414	47.73	SMILE + RT
UPN38	61	0	NA	extranodal NKTL	57.65%	4.703871	1.06399	29.87056	0.50706	23.5	SMILE+Pembrolizumab
UPN39	37	0	Chinese	extranodal NKTL	59.89%	5.002218	0.584317	30.39401	0.218481	NA	SMILE + RT
UPN40	43	1	Chinese	extranodal NKTL	57.12%	4.991527	0.699007	28.887	0.189985	NA	NA
UPN41	60	1	Chinese	extranodal NKTL	87.00%	6.773904	0.507205	30.66179	0.147808	6.87	LVP + RT
UPN42	69	1	Malay	extranodal NKTL	70.59%	3.817556	0.881828	23.60274	0.38975	NA	NA

Abbreviations:

UPN: unique patient number

NA: information not available

LVP: L-asparaginase, vincristine, and prednisone

L-ASP: L-asparaginase

ETP: etoposide

FLU: fludarabine

VCR: vincristine

PSL: prednisolone

HCT: hydrocortisone:

CHOP: cyclophosphamide, doxorubicin (hydroxydaunomycin), vincristine (oncovin), and prednisolone

DHAP: dexamethasone, cisplatin, cytarabine

SMILE: dexamethasone, methotrexate, ifosfamide, L-asparaginase, and etoposide

GIFOX-B: gemcitabine, ifosfamide, oxaliplatin, and bortezomib

m-BACOD: methotrexate, bleomycin, doxorubicin (adriamycin), cyclophosphamide, vincristine, and dexamethasone

ICE: ifosfamide, carboplatin, and etoposide

CT: chemotherapy

RT: radiation therapy

Supplemental figures

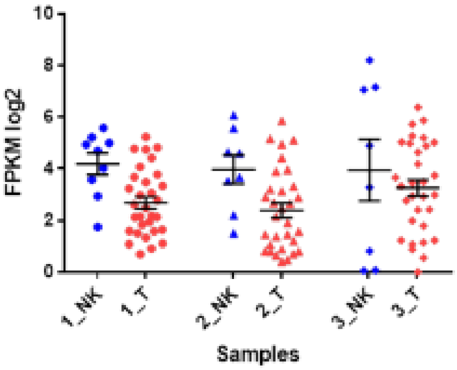

Index	Sample	Xiong_NK.Tsig (higher = T-cell origin)_p	Xiong_NK.Tsig (higher = T-cell origin)_score
1	NKTL4	0.015	-0.5
2	NKTL9	0.044	-0.43
3	NKTL10	0.068	-0.4

Figure S1. Determination of cell-origin of 3 NKTL cases on their RNA-seq data. This analysis was performed by using a two-sample Kolmogorov-Smirnov based method developed in house and the NK cell and T cell signature published by Xiong J, et al (Cancer Cell. 2020 Mar 16;37(3):403-419). This dot plot shows the genes expression of NK-origin (blue) and T-cells-origin (red). Lowly expressed genes (mean FPKM < 1) has been filtered out.

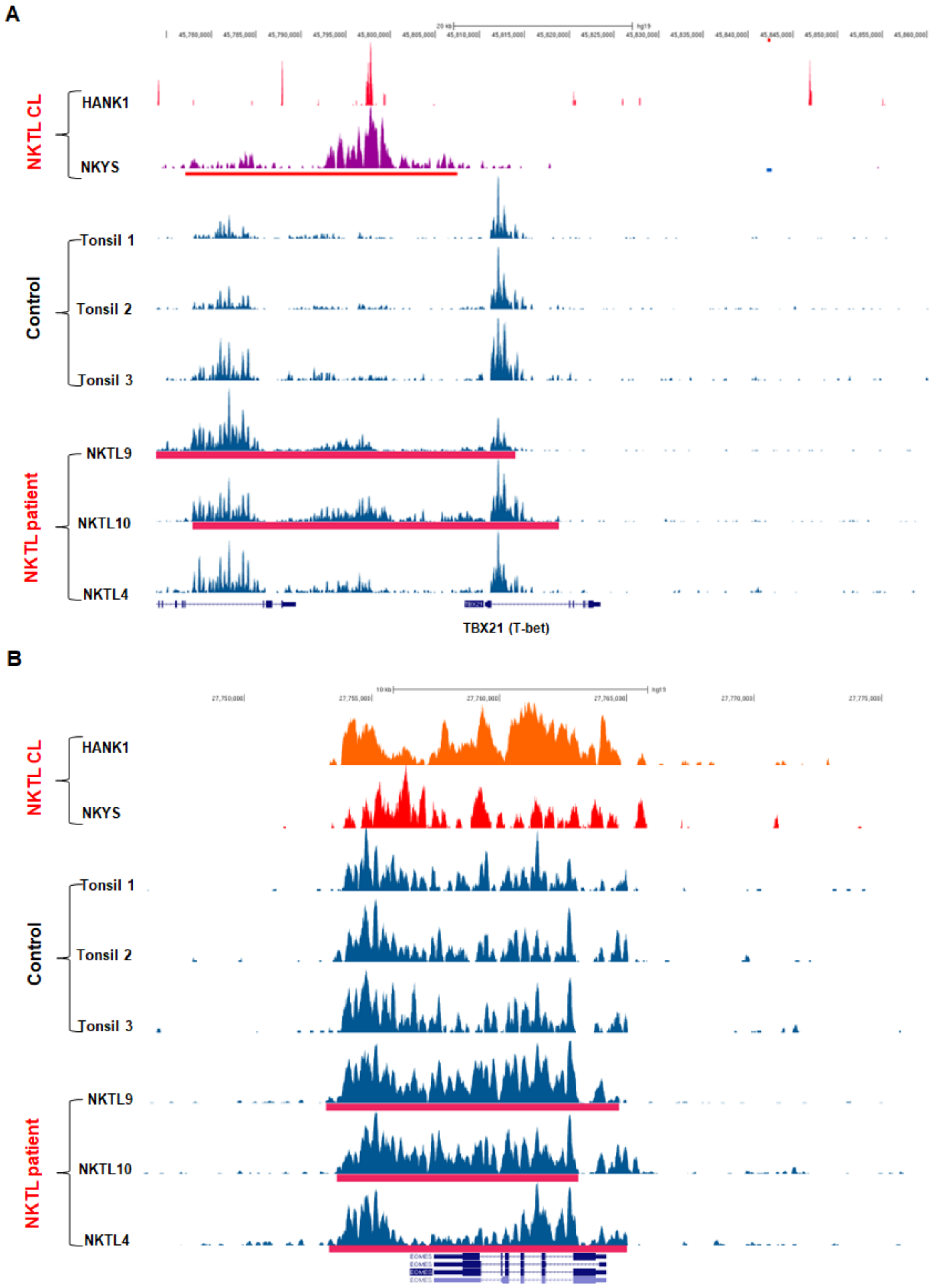

Figure S2. UCSC Genome Browser ChIP-Seq screenshot. Track view of H3K27ac ChIP-seq density profile centered at the TBX21 (T-bet) and EOMES gene loci of NKTL cell line HNAK1 and NKYS (top panel), 3 tonsil controls (middle panel) and 3 primary NKTL patient samples (lower panel). Locations of the SEs regions were marked by red bars.

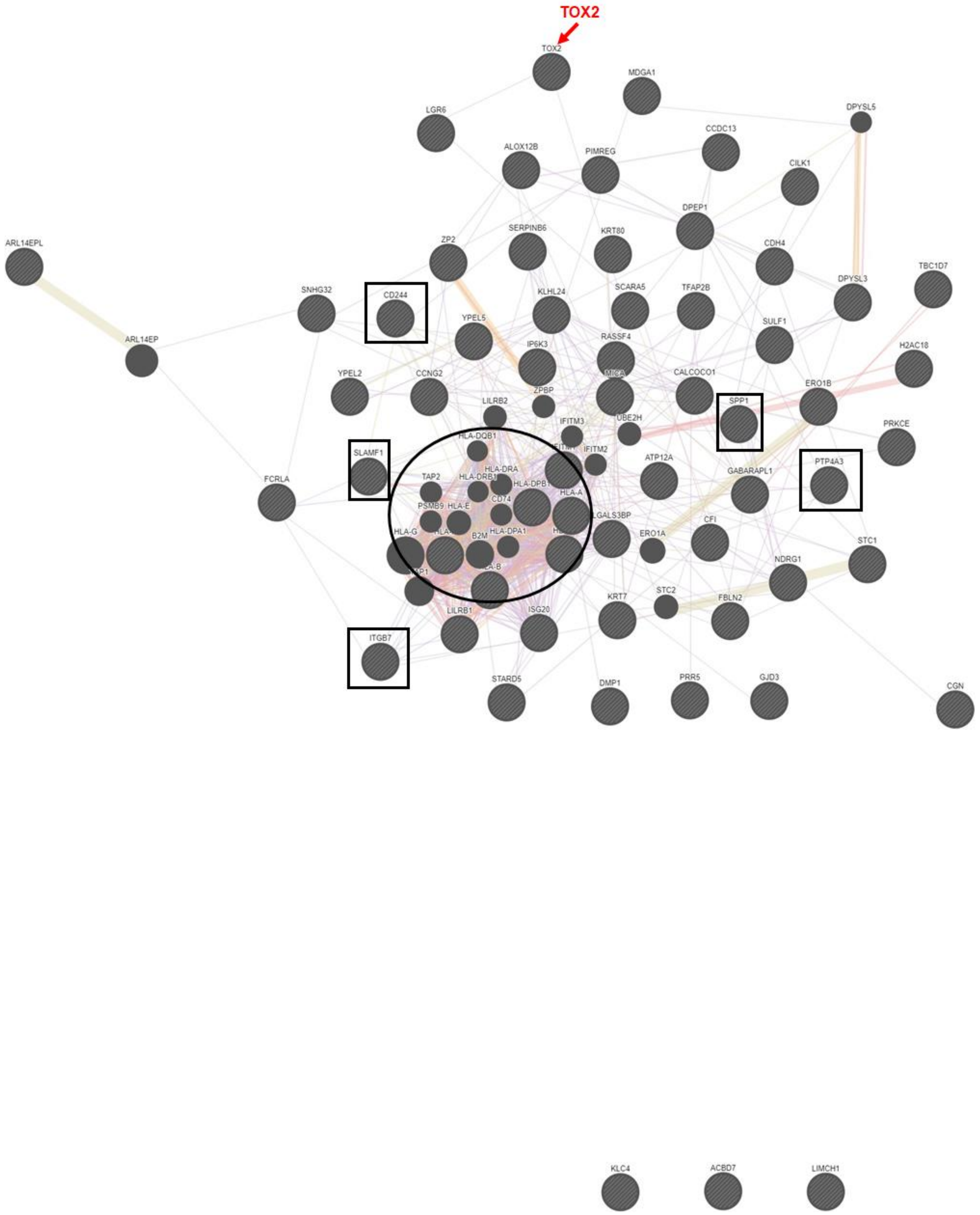

Figure S3. Gene network of NKYS cells responding to TOX2 knockdown derived from GeneMANIA. A gene network from GeneMANIA shows the relationships for genes from the list of downregulated genes induced by TOX2-shRNAs according to the functional association networks from the databases. TOX2 sat on the top of the network and a physical interaction of network, indicated by a red arrow. Black rectangles highlight a few important targets, including PTP4A3, SPP1, SLAMF1, CD244, ITGB7. A black circle comprises some MHC family members.

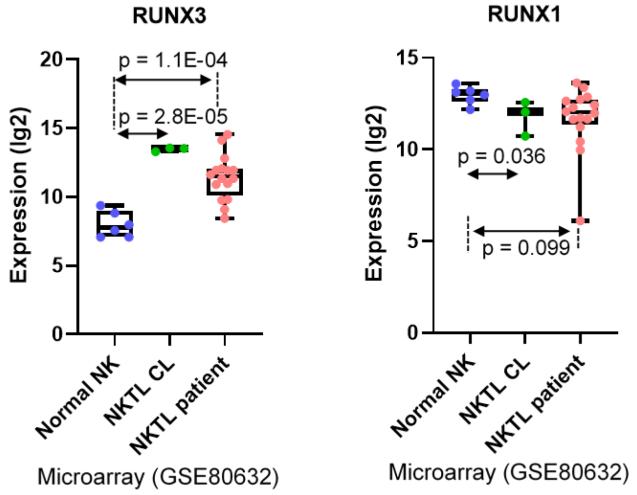

Figure S4. The expression of RUNX1 and RUNX3 in normal NK cells, NKTL cell lines and NKTL patient samples. Expression (lg2) level of RUNX1 and RUNX3 in a collection of normal NK cells, NKTL cell line and NKTL patient samples derived from a microarray dataset in Gene Expression Omnibus (GEO) database (accession number: GSE80632). p value < 0.05 is considered as statistically significant.

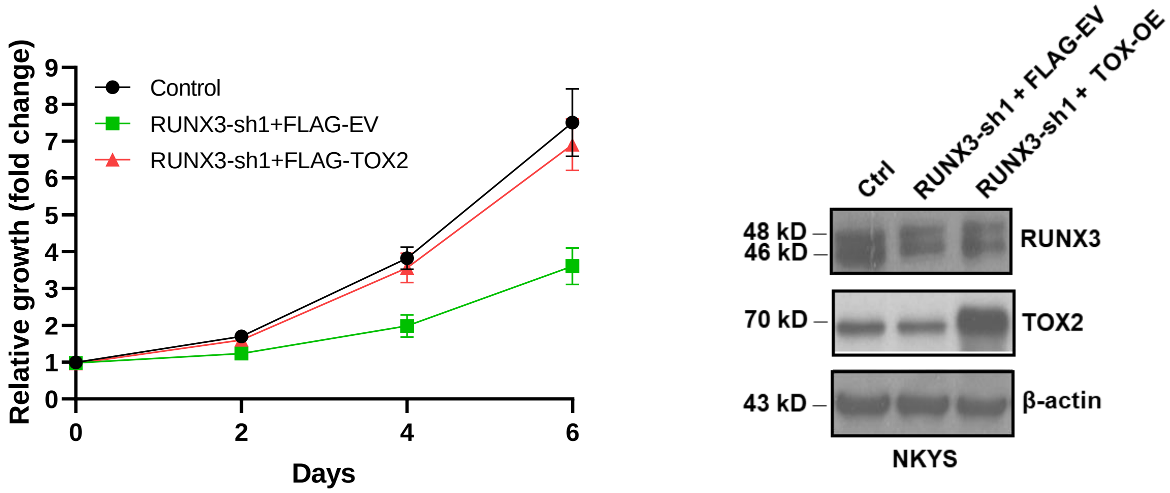

Figure S5. TOX2 overexpression rescues NKYS cells from RUNX3 depletion. (A) Comparison of the rate of growth of co-transduced NKYS cells expressing FLAG-EV (empty vector) or FLAG-TOX2 and RUNX3-sh1 constructs. NKYS cells co-transduced with FLAG-EV and scramble shRNA were used as control. For each condition, cell number was counted at day 2, 4, and 6, then converted to fold change relative to the starting number at day 0. Same number of cells were seeded at day 0 and comparison was made at indicated days for relative fold changes. Three biologically independent experiments were performed (mean ± SD). Western blot analysis showing the expression of RUNX3 and TOX2 shown on the right. β-actin was used as the loading control.
